# Sequential release of *N*-butylphthalide via multifunctional hydrogel for rapid neuroprotection and sustained neural repair after traumatic brain injury

**DOI:** 10.1016/j.mtbio.2026.103436

**Published:** 2026-07-06

**Authors:** Changbin Hu, Ning Li, Yuanyuan Ran, Hao Fu, Lei Zhou, Fanglei Li, Chenye Qiao, Chuhan Liu, Guiqin Tian, Guoping Guan, Wei Su, Jianing Xi, Zongjian Liu

**Affiliations:** a Beijing Rehabilitation Hospital, Capital Medical University, Beijing, 100144, PR China; b College of Textiles, Donghua University, Shanghai, 201620, PR China; c Beijing Tsinghua Chang Gung Hospital, School of Clinical Medicine, Tsinghua University, Beijing, 102218, PR China

**Keywords:** Traumatic brain injury, N-butylphthalide, Hydrogel, Microglial polarization, Ferroptosis

## Abstract

Timely neuroprotective therapy in the acute phase, combined with sustained neural repair strategies in the subacute and chronic phases, is crucial for functional recovery following traumatic brain injury (TBI). However, few pharmaceutical interventions currently achieve cross-phasic modulation with a single administration. Thus, developing a drug delivery system with sequential neuroprotective and neural repair capabilities is urgently required. Herein, we fabricated an injectable multifunctional methacrylated alginate hydrogel integrated with cyclodextrin inclusion complexes (to improve the poor solubility of n-butylphthalide, NBP) and a sucrose acetate isobutyrate (SAIB) depot (to extend retention and enable delayed release). A single implantation of this hydrogel into the post-TBI cavity exerted sequential therapeutic effects: it rapidly released NBP to mitigate ferroptosis in the acute phase, subsequently regulated microglial polarization via sustained release, and ultimately enhanced neural plasticity in the late stages in a mouse model. We employed clinical database analysis, mouse transcriptome analysis, and in vivo experiments, which identified ferroptosis as a key driver of the pathophysiological process of TBI. Further network pharmacology, molecular docking, and in vitro experiments demonstrated that NBP attenuated TBI-induced ferroptosis through the GSK-3β-Fyn-Nrf2 pathway. Collectively, our work provides a multi-stage therapeutic platform with controlled NBP release for TBI intervention.

## Graphical abstract

**Figure:**
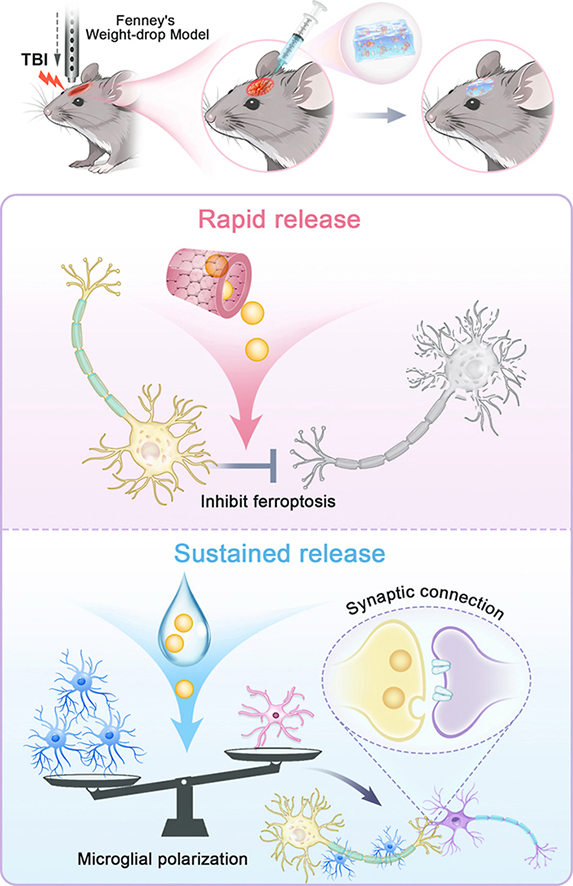

## 1 Introduction

**Scheme 1:**
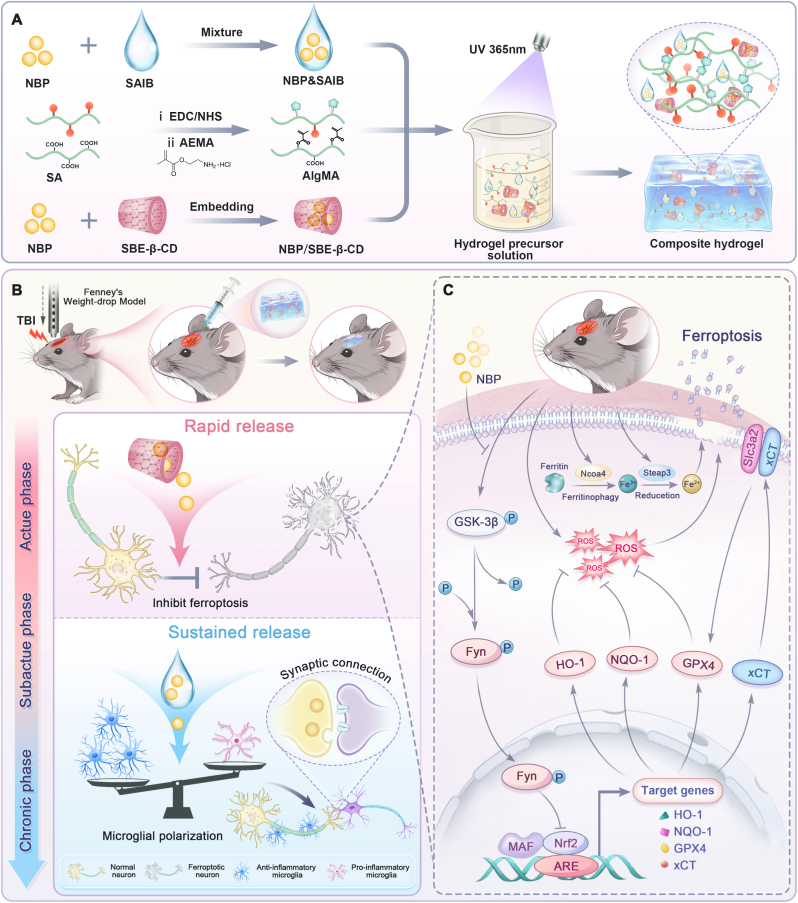
Preparation of NBP-loaded hydrogel (A) and its sequential release property enabling rapid neuroprotection and sustained neural repair after TBI. The multifunctional hydrogel rapidly released NBP to mitigate ferroptosis and oxidative damage in the acute phase, subsequently regulated microglial polarization via sustained release, and ultimately enhanced neural plasticity in the late stages (B). Mechanistically, NBP attenuated TBI-induced ferroptosis via the GSK-3β-Fyn-Nrf2 signaling pathway (C).

TBI remains a major global public health issue and is one of the leading causes of death and long-term disability across all age groups [1,2]. Many survivors do not fully recover and still have cognitive, emotional, and motor problems that make it hard for them to return to daily life independently. These problems put a lot of stress on families and healthcare systems [3]. Despite significant advances in TBI management, such as refined intracranial pressure monitoring, optimized decompressive surgery, standardized analgesia and sedation protocols, and improved maintenance of cerebral perfusion and oxygenation, clinical outcomes following TBI continue to demonstrate considerable variability. Current treatment strategies are primarily supportive, and there remains an absence of disease-modifying pharmacotherapies that can effectively halt the pathological progression of TBI [4]. These challenges underscore a key therapeutic dilemma: TBI evolves dynamically over time, with early treatment focusing on neuron protection during the acute phase. As the injury progresses to the subacute and chronic stages, therapeutic strategies increasingly shift toward promoting microenvironmental remodeling and neuroplasticity [[5], [6], [7]]. However, conventional drug-dependent therapeutic approaches often fail to achieve the necessary temporal matching between drug exposure and stage-specific therapeutic needs (see Scheme 1).

In the acute phase of TBI, neuron death and secondary injury cascades dominate the pathological landscape. Neuron protection is the primary therapeutic goal. The secondary injury cascade involves oxidative stress, mitochondrial dysfunction, the inflammatory cascade, and many forms of cell death, such as apoptosis and necrosis. These events are highly intertwined and progressively amplified. Together, they ultimately result in massive neuronal loss and irreversible impairment of brain function. After TBI, disruption of the blood-brain barrier (BBB) leads to abnormal iron accumulation in brain tissue. At the same time, intracellular iron storage and trafficking pathways become dysregulated. Excess iron then catalyzes the generation of reactive oxygen species (ROS) through Fenton chemistry and triggers chains of lipid peroxidation. As a result, membrane integrity is compromised, leading to ferroptotic neuronal death. This mechanism provides an important entry point for targeted interventions during the acute stage of TBI. Notably, iron metabolism dysregulation and ferroptosis have emerged as critical contributors to acute neuron loss after TBI [].

Based on the pathological mechanisms in the acute phase of TBI, targeted therapeutic strategies primarily focus on: (A) Inhibiting ferroptosis and prevent damage from lipid peroxidation; (B) Using antioxidants to activate the Nuclear factor erythroid 2-related factor 2 (Nrf2) pathway and remove harmful ROS; (C) Protecting the mitochondria and adjust metabolism; and (D) Applying anti-inflammatory and immunomodulatory therapies. During the subacute-to-chronic phase, treatment focus shifts from limiting injury to promoting repair and remodeling. At these stages, spontaneous repair programs are initiated, and recovery increasingly depends on neuroplasticity, despite persistent barriers such as restricted axonal regeneration, glial scar formation, and an ongoing pro-inflammatory microenvironment [8,9]. Microglial polarization is crucial for regulating synaptic remodeling and plasticity, while broader microenvironmental reprogramming further shapes circuit reorganization and functional recovery [10]. Therefore, subacute and chronic treatment strategies focus on enhancing plasticity, synaptic remodeling, and neurogenesis. Additionally, these strategies aim to modulate the immune microenvironment by promoting repair-associated phenotypes and reducing chronic inflammation [11]. An ideal TBI therapy should address both phases by providing acute neuroprotection, such as targeting ferroptosis, and facilitating subacute and chronic repair through mechanisms like immunomodulation and neuroplasticity. This highlights the need for a time-programmed delivery approach. NBP is a neuroprotective drug with broad biological activities, including antioxidative effects, mitochondrial protection, anti-inflammatory actions, and improvement of microcirculation [[12], [13], [14]]. Importantly, these properties are highly relevant to our mechanistic emphasis on injury related to iron metabolism in the acute phase and the potential for neuroplasticity during subacute and chronic phases. However, in both preclinical and translational studies, NBP is most commonly administered systemically [15]. While systemic dosing may be feasible during the acute window when BBB permeability is transiently increased after TBI, it becomes increasingly difficult to maintain therapeutically effective intracerebral exposure as the BBB progressively recovers and peripheral metabolism reduces brain availability in the subacute-to-chronic phases [16,17]. Therefore, constructing a biomaterial local drug delivery system for targeted brain delivery and time-controlled release of NBP has become a rational approach to address this challenge. It is also a critical direction for overcoming the therapeutic dilemmas of TBI in the field of medicine and engineering. In this context, recent studies have shown that functional biomaterial-based strategies can provide favorable regenerative microenvironments and enhance tissue regeneration and functional repair after brain injury [18,19].

Injectable hydrogels are hydrophilic polymer materials with three-dimensional network structures that show significant promise for neural injury treatment. Their main benefits include excellent biocompatibility, the ability to gel in situ, and efficient drug-loading. They quickly form a scaffold that matches brain tissue's microenvironment, supporting tissue compatibility [20]. These hydrogels can encapsulate various therapeutic carriers-including small-molecule drugs, proteins, exosomes, and cells-while allowing controlled release of treatment factors and supporting local microenvironment modulation. Importantly, the formation of a post-traumatic cavity after TBI provides a natural space and structural basis for in situ biomaterial implantation and filling, creating a distinct advantage that makes localized delivery a feasible and rational therapeutic strategy by enabling precise drug administration at the lesion site while minimizing systemic exposure and improving intracerebral bioavailability. [21]. Substantial progress has been made in applying hydrogels for neural injury repair, with major applications primarily falling into three categories: (i) drug-loaded hydrogels delivering anti-inflammatory, antioxidative, or neurotrophic agents-exemplified by injectable hydrogel systems developed in ischemic stroke that combine therapeutic delivery with microenvironment regulation, such as a curcumin-loaded hydrogel with dual ROS-scavenging capability to regulate microglial polarization and promote post-stroke functional recovery [22], as well as a multifunctional injectable hydrogel that enhances recovery by modulating microglial polarization, angiogenesis,and neuroplasticity [23]; (ii) exosome-based hydrogels for modulating immune responses and promoting repair signaling [24]; and (iii) hydrogels loaded with cells or scaffold-based approaches that provide structural integrity and support a regenerative microenvironment [25]. In addition, our group has extended and evaluated hydrogel-based delivery in TBI models, demonstrating the feasibility of an in situ implantable, ROS-responsive hydrogel loaded with minocycline to support functional rehabilitation after TBI [24]. Despite these advances, most current designs emphasize either sustained release or a single-mode release profile, and thus remain insufficiently aligned with the stage-dependent therapeutic demands of TBI. There is still a lack of systematic design and validation of biphasic (burst plus sustained) release systems that provide early neuroprotection and prolonged, repair-oriented modulation, which represents a critical gap that our work aims to fill.

In this study, we used the implantable post-traumatic cavity as a unique delivery site and develop an injectable hydrogel platform for localized NBP delivery. The system achieves a biphasic release profile by combining burst release during the acute phase and sustained release during the subchronic phase. During the acute phase, rapid NBP release targets the ferroptosis pathway and oxidative stress response. By blocking secondary injury cascades, it achieves rapid neuroprotection. In the subchronic phase, sustained NBP release modulates microglial polarization direction over the long term. It optimizes the local repair microenvironment and alleviates the effects of chronic glial inflammation on neural plasticity. This promotes synaptic remodeling and neural functional reconstruction. To enable this therapeutic strategy, this study developed a hierarchical, staged-release composite hydrogel. It integrates biodegradable drug-loaded hydrogels for acute-phase NBP burst release and utilizes SAIB for sustained subchronic-phase drug release. Additionally, a degradation-release synergistic mechanism was established to ensure precise alignment between drug release rates and the pathological progression of TBI. This research not only developed a clinically translatable NBP-loaded composite hydrogel, but also established a programmable sequential release design paradigm for dynamic TBI pathological processes. It provides a framework for developing stage-adaptive neurotherapeutic delivery systems.

## 2 Materials and methods

### 2.1 Clinical data analysis

Clinical data of patients with TBI were obtained from the Medical Information Mart for Intensive Care (MIMIC-IV) database. Patients were included based on a diagnosis of TBI and availability of baseline serum ferritin measurements. Patients were stratified into groups according to ferritin levels. Kaplan–Meier survival analysis was performed to compare survival outcomes between groups, and differences were assessed using the log-rank test. Time-dependent receiver operating characteristic (ROC) curve analysis was conducted to evaluate the prognostic performance of ferritin across different follow-up periods.

### 2.2 Mendelian randomization analysis

Two-sample Mendelian randomization (MR) analysis was performed to assess the causal association between circulating micronutrients and TBI risk. Genetic instruments for serum micronutrients were obtained from publicly available genome-wide association study (GWAS) summary statistics of European ancestry populations, with data available for 15 micronutrients from the GWAS database. Single-nucleotide polymorphisms (SNPs) associated with serum micronutrient levels at genome-wide significance were selected as instrumental variables after linkage disequilibrium clumping. Summary-level GWAS data for TBI were obtained from the FinnGen consortium. MR analyses were conducted using the inverse-variance weighted (IVW) method and MR-Egger regression. Sensitivity analyses were performed using a leave-one-out approach to assess the influence of individual SNPs on the overall MR estimates.

### 2.3 Transcriptomic data analysis and gene set enrichment analysis

Publicly available transcriptomic data from a mouse traumatic brain injury model (GSE79441) were downloaded from the Gene Expression Omnibus (GEO) database. Raw expression data were processed and normalized using standard bioinformatics pipelines. Differentially expressed genes between TBI and control samples were identified using the limma package in R. Gene set enrichment analysis (GSEA) was performed using the GSEA R package. Ranked gene lists derived from differential expression analysis were analyzed against curated gene sets related to ferroptosis, regulation of ferroptosis, iron ion transport, and iron metabolism. Enrichment scores and normalized enrichment scores were calculated, and gene ontology(GO) biological process enrichment was summarized using bubble plots based on enrichment significance and gene counts.

### 2.4 Network pharmacology, PPI network, and enrichment analysis

Drug-target interaction data for NBP were retrieved from the SwissTarget and SEA databases, and TBI-related targets were obtained from OMIM and GeneCards. The common targets between NBP and TBI were identified by intersecting the corresponding target lists. A protein–protein interaction (PPI) network of the intersected targets was constructed using the STRING database and visualized with Cytoscape. GO enrichment analysis and Kyoto Encyclopedia of Genes and Genomes (KEGG) pathway enrichment analysis were conducted using the DAVID database to characterize the associated biological processes, cellular components, molecular functions, and pathways of the common targets.

### 2.5 Molecular docking

Molecular docking was performed to evaluate the binding interaction between NBP and selected key target proteins. The three-dimensional structure of NBP was obtained from the PubChem database, and the crystal structures of target proteins were downloaded from the Protein Data Bank (PDB). Protein structures were prepared using PyMOL 3.0 by removing water molecules and adding polar hydrogens. Ligands and protein targets were processed using AutoDockTools 1.5.7 to assign charges and define docking grids. Docking simulations were conducted with AutoDock Vina 1.1.2 using standard protocols. The resulting docking conformations were analyzed for binding interactions using LigPlot + software, and final docking poses were visualized with PyMOL 3.0.

### 2.6 Synthesis of SAIB&NBP and NBP@CD

A homogeneous mixture of SAIB and NBP (8:1, v/v) was obtained by vigorous magnetic stirring for 4 h, yielding the final SAIB-NBP blend solution. A solution of 0.4 g sulfobutyl ether-β-cyclodextrin (SBE-β-CD) in 20 mL deionized water was prepared under constant stirring in a water bath maintained at 67°C. Separately, 0.0197 g n-butylphthalide (NBP) was dissolved in 2 mL anhydrous ethanol. The NBP solution was added dropwise to the SBE-β-CD solution under agitation, with a molar ratio of SBE-β-CD to NBP fixed at 2.6: 1.5. The mixture was shaken for 2 h at 67°C, followed by continuous stirring during cooling to room temperature. Unencapsulated NBP was removed by filtration through a 0.45 μm microporous membrane. The final inclusion complex was obtained by freeze-drying the filtrate.

### 2.7 Synthesis of AlgMA

Methacrylated alginate (AlgMA) was synthesized via carbodiimide-mediated coupling of 2-aminoethyl methacrylate (AEMA) to the carboxyl groups of sodium alginate. Briefly, sodium alginate (2.5 g) and 2-morpholinoethyl sulfonic acid (MES; 2.44 g, 50 mM) were dissolved in 250 mL deionized water under stirring at room temperature and the pH of the solution was adjusted to 5–6 using 5 M NaOH. The carboxyl groups of alginate were activated by sequential addition of N-hydroxysuccinimide (NHS; 1.035g) and 1-(3-dimethylaminopropyl)-3-ethylcarbodiimide hydrochloride (EDC; 2.79 g), followed by reaction for 15 min under gentle agitation. Subsequently, AEMA (1.5 g) was introduced, and the reaction was allowed to proceed in the dark for 24 h. The product was precipitated by adding the reaction mixture into a large excess of anhydrous ethanol, recovered by centrifugation, and the product was evaporated in a fume hood. The resulting solid was redissolved in 250 mL deionized water and purified by dialysis (MWCO 3500) against deionized water for 3 days. The final product (AlgMA) was obtained as a white powder after lyophilization.

### 2.8 Preparation of NBP/CD@Gel/NBP&SAIB@Gel/NBP@Gel

Three aliquots of 1.0% (w/v) AlgMA solution were prepared by dissolving AlgMA in deionized water. To each aliquot, an appropriate amount of inclusion complex, SAIB-NBP mixture, or a combination of both (corresponding to the Fast, Slow, and Composite formulations, respectively) was added. Subsequently, 0.25% (w/v) of the photoinitiator Irgacure 2959 (I2959) was incorporated into each solution. A defined volume of each mixture was then transferred into separate silicone molds and photo-crosslinked by exposure to UV light for 5 min to form the primary hydrogel network.

### 2.9 Characterizations

The chemical functionalization of AlgMA was verified with 1H nuclear magnetic resonance (NMR, AV600 MHz, Bruker, Switzerland). The chemical structure and composition of different samples were analyzed by Fourier transform infrared spectroscopy (FTIR, Spectrum Two, PerkinElmer, USA).

### 2.10 Pore size, and biodegradation tests

The pore size of the lyophilized samples was observed by the corresponding SEM images, and the mean pore size was analyzed by ImageJ software. The in vitro degradation test of different samples was performed as follows. The initial weight of lyophilized samples was recorded as Wa, and then these samples were placed in PBS at 37°C with constant shaking at 60 rpm. At different time points, the samples were removed and lyophilized to obtain a constant weight (recorded as Wb). The in vitro degradation rate was calculated according to the following formula: weight remaining (%) = Wb/Wa × 100%.

### 2.11 Rheological testing

The rheological properties of NBP/CD@Gel, NBP&SAIB@Gel, NBP@Gel were characterized with a rotational Rheometer (MCR302e,Anton Paar, Austria). Strain sweeps were applied with strain ranging from 0.1% to 100% and a fixed oscillation frequency of 1 rad/s. Frequency sweeps were applied with oscillation frequencies from 0.1 to 100 rad/s and a fixed strain of 1%.

### 2.12 In vitro release profile of NBP from different hydrogels

In vitro, drug release assays were detected with a UV-Visible Spectrophotometer. The in vitro drug release profile of the hydrogel loaded with 1432.9 μg of NBP was characterized as follows: The hydrogel was individually immersed in 10 mL of pre-warmed PBS within centrifuge tubes. The tubes were incubated in an orbital shaker maintained at 37°C and agitated at 60 rpm. At predetermined time intervals, 3 mL of the release medium was collected and simultaneously replaced with an equal volume of fresh, pre-warmed PBS to maintain sink conditions. The cumulative amount of NBP released was calculated based on the concentrations measured in the withdrawn samples.

### 2.13 Animals

Adult male C57BL/6J mice (8 weeks old, 24.0 ± 1.5 g) were purchased from Beijing Vital River Laboratory Animal Technology Co., Ltd. (Beijing, China). Animals were provided food and water ad libitum and housed under controlled temperature (23 ± 2°C) and humidity (40–60%) with a 12-h light/dark cycle, and acclimated for 7 days before experiments. Animals were randomly assigned to experimental groups, and all outcome assessments were performed with investigators blinded to treatment. All in vivo procedures were approved by the Animal Ethical Committee of Beijing Rehabilitation Hospital, Capital Medical University (approval ID: 2024bkky-087).

### 2.14 TBI model (Feeney weight-drop)

Mice were anesthetized with isoflurane (2% induction,1.5% maintenance), the scalp was shaved and disinfected, and animals were fixed in a stereotaxic frame (RWD, Shenzhen). A midline scalp incision was made to expose the skull. A craniotomy was created 2 mm left of the midline and 3 mm posterior to bregma using a 4 mm diameter trephine; the dura was kept intact. A 2.5 mm impactor tip was centered in the bone window and lowered until just touching the dura, then the vertical scale was advanced 3.0 mm. A 40 g weight was released from 7.5 cm to strike the firing pin and produce moderate TBI in accordance with the Feeney method [26]. Mice were assigned to seven groups: Sham (craniotomy only), TBI, TBI + Gel, TBI + NBP, TBI + NBP@Gel, TBI + NBP/CD@Gel (immediate-release component only), and TBI + NBP&SAIB@Gel (sustained-release component only). Immediately after debridement, 7 μL of the corresponding formulation was injected into the lesion cavity. The scalp was sutured, and mice were maintained on a 37°C heating pad for 10 min until recovery, then returned to clean cages with food and water ad libitum.

### 2.15 Behavioral assessment

Mice were trained for 3 days before baseline testing. Behavioral tests were performed on days 1, 3, 7, and 14 post-TBI. Rotarod (Panlab LE8505): speed accelerated from 4 to 25 rpm in 3 min; latency to fall was recorded. Beam walk: mice traversed an 80 cm long, 1.5 cm wide beam elevated 100 cm above the floor; each slip of the right hind paw was counted as one falling event for statistical analysis. If a mouse fell off the beam, it was returned to the point at which it fell and continued the trial. Novel Object Recognition (NOR) was performed on day 14 after TBI. Mice were habituated to the empty chamber for 10 min, then exposed to two identical objects for 5 min. After 1 h, one object was replaced with a novel object and exploration was recorded for another 5 min. The recognition index was calculated as novel object exploration time/total exploration time ∗ 100%.

### 2.16 In vivo fluorescence tracking

Rhodamine B was dissolved in PBS to prepare Rhodamine solution (Rho solution), and mixed with the hydrogel to prepare Rhodamine-loaded hydrogel (Rho-Gel). These formulations were implanted into the lesion site of mice following TBI. Fluorescence signals were monitored using a small-animal imaging system (IVIS® Lumina III, PerkinElmer, USA) at 1, 3, 7, and 14 days post-injury. Signal persistence and distribution at the lesion site were compared between Rho Solution and Rho-Gel groups.

### 2.17 Western blot

Peri-lesional cortical tissues were rapidly dissected, washed with ice-cold PBS, and homogenized on ice in RIPA buffer freshly supplemented with protease and phosphatase inhibitors. Cultured cells were washed with ice-cold PBS and lysed on ice in the same RIPA buffer. Lysates were clarified (12,000 g, 10 min, 4°C), and protein concentration was determined using a BCA assay kit (Thermo Fisher Scientific, USA). Equal amounts of protein (30 μg) were denatured, separated by SDS-PAGE, and transferred onto PVDF membranes. Membranes were blocked with 5% dry milk for 1h at room temperature, and incubated with primary antibodies overnight at 4°C. Antibody Information: STEAP3 (Proteintech, 28478-1-AP), NCOA4 (Abcam, ab314553), xCT (Abcam, ab307601), ACSL4 (Abcam, ab155282), NRF2 (Cell Signaling Technology, 12721S), HO-1 (Cell Signaling Technology, 43966S), NQO1 (Cell Signaling Technology, 3187S), GPX4 (Abcam, ab125066), COX2 (Cell Signaling Technology, 12282S), p-GSK3β (Ser9) (Cell Signaling Technology, 5558S), Fyn (Cell Signaling Technology, 4023S), PSD95 (Abcam, ab2723), VGLUT1 (Abcam, ab180188), and GAPDH (Cell Signaling Technology, 5174S). After primary antibody incubation, membranes were washed and then incubated with HRP-conjugated secondary antibodies (Abcam or Solarbio) for 1h at room temperature, followed by visualization using enhanced chemiluminescence. Band intensities were quantified using ImageJ software and normalized to GAPDH.

### 2.18 Oxidative-stress assays

Cells or peri-lesional brain tissues were processed on ice. Tissues were homogenized in pre-chilled normal saline to obtain a 10%(w/v) homogenate using a glass homogenizer. Cell samples were collected and prepared as lysates in the buffer specified by the assay kits. The lysates were centrifuged at 12,000×g for 15 min at 4°C, and the supernatants were collected for downstream assays. Total protein concentration was determined and used for normalization of all readouts. ROS levels were measured using a DCFH-DA–based ROS assay kit (Solarbio, CA1410). Lipid peroxidation was assessed by measuring malondialdehyde (MDA) using an MDA assay kit (Solarbio, BC0025). Superoxide dismutase (SOD) activity was determined using a SOD assay kit (Solarbio,BC5165). Catalase (CAT) activity was measured using a CAT assay kit (Solarbio, BC0205). All protocols were performed according to the manufacturer's instructions.

### 2.19 ELISA for cytokines

Supernatants prepared as above were further clarified by centrifugation at 12,000×g for 10 min at 4°C to remove debris. IL-10 and TGF-β levels were quantified using ELISA kits (Shanghai Jianglai Bio, IL-10: JL20242; TGF-β: JL13959) according to the manufacturer's protocols. Samples were diluted prior to loading (IL-10, 1: 10; TGF-β, 1: 5). Standard curves were prepared using serial dilutions of the provided standards. For each well, 100 μL standards or diluted samples were added, incubated at 37°C for 2h, washed, followed by incubation with detection antibody (37°C,1 h) and HRP conjugate (37°C,1 h) with the indicated wash steps between incubations. After color development with substrate solution (37°C, 15min, in the dark), the reaction was stopped and absorbance was read at 450 nm. Cytokine concentrations were calculated from the standard curves and normalized to protein content, reported as pg/mg protein.

### 2.20 Immunofluorescence

Cells and brain tissues were processed for immunofluorescence staining. For cell staining, cells were cultured on poly-L-lysine–coated coverslips (0.1 mg/mL). After treatment, cells were fixed with 4% paraformaldehyde for 20 min, permeabilized with 1% PBST for 15 min at room temperature, and blocked with 10% donkey serum in PBS for 2 h. Cells were then incubated with primary antibodies diluted in 0.3% PBST overnight at 4°C, washed with 0.3% PBST, and incubated with fluorophore-conjugated secondary antibodies (Abcam or Solarbio) diluted in 0.3% PBST for 1 h at room temperature in the dark. For brain tissue staining, peri-lesional cortical sections were prepared, permeabilized and blocked as above, then incubated with the same primary/secondary antibody workflow. Nuclei were counterstained with DAPI, and sections were mounted with DAPI Fluoromount-G (SouthernBiotech, 0100–20). Images were acquired using a Nikon A1 confocal microscope. Fluorescence intensity and positive-cell quantification were analyzed using ImageJ software. Primary antibodies used were as follows: iNOS (Abcam, ab178945), CD206 (Abcam, ab64693), PSD95 (Abcam, ab18258), and VGLUT1 (Abcam, ab180188).

### 2.21 H&E staining

Brain tissues and other major organs-including the heart, liver, spleen, lungs, and kidneys were dehydrated through graded ethanol (50-100%; Sinopharm, Cat.No. 10009218), cleared in xylene (Sinopharm, Cat.No.10023418), paraffin-embedded, and sectioned at 5 μm using a microtome (Leica RM2235). Sections were mounted on poly-L-lysine–coated slides (Solarbio, Cat.No.SL016) and dried (Leica HI1220), then deparaffinized in xylene and rehydrated through descending ethanol to water. H&E staining was performed with hematoxylin (Solarbio, Cat.No.G1080) followed by differentiation/bluing and eosin counterstaining (Solarbio, Cat.No.G1120). After staining, sections were dehydrated, cleared in xylene, and coverslipped with neutral resin mounting medium (Solarbio, Cat.No.G8590) and coverslips (Thermo Scientific, Cat.No.12-550-15).

### 2.22 Nissl staining

Paraffin-embedded brain sections were mounted on slides, dried, deparaffinized and rehydrated. Nissl staining was performed using toluidine blue solution (Solarbio, Cat.No.G1430). Sections were incubated in toluidine blue at 37°C for 10 min, rinsed in distilled water, and differentiated in 0.5% acetic acid until background staining was reduced. Sections were then dehydrated through graded ethanol, cleared in xylene, and mounted with neutral resin medium and coverslips. Images were captured under a light microscope, and Nissl-positive neurons were counted in the peri-lesional cortex.

### 2.23 Immunohistochemistry

Paraffin-embedded mouse brain sections were mounted on slides, and baked at 60°C for 2 h. Sections were deparaffinized in xylene and rehydrated through a graded ethanol series to distilled water. After PBS rinses, endogenous peroxidase activity was quenched with 3% H_2_O_2_ for 10 min, followed by PBS washes. Antigen retrieval was performed in citrate buffer (pH 6.0) using microwave heating, and sections were cooled to room temperature. Non-specific binding was blocked with goat serum at 37°C for 30 min. Sections were incubated with 4-HNE (Servicebio, GB150073) overnight at 4°C, followed by incubation with HRP-conjugated goat anti-rabbit IgG for 30 min at 37°C. After washing, DAB development was performed under microscopic monitoring. Nuclei were counterstained with hematoxylin, differentiated in 1% acid alcohol,and blued in running water.

### 2.24 Blood biochemical and hematological analysis

Peripheral blood samples were collected from mice on day 14 after treatment for biosafety evaluation. Part of the blood sample was used for routine hematological analysis, and the remaining sample was centrifuged at 3000 rpm at 4°C for 5 min to collect the supernatant for biochemical analysis. Serum levels of ALT, AST, ALB, and LDH were measured using an automatic biochemical analyzer (Hitachi 7180, Japan) according to the manufacturer's instructions. Hematological parameters, including RBC, WBC, HGB, MCH, and MCHC, were determined using an automated hematology analyzer (Sysmex XT-2000i, Japan) in peripheral blood mode according to standard procedures.

### 2.25 Preparation of gel extract and NBP@Gel extract

To prepare Gel extract, the blank hydrogel (Gel) was incubated in complete culture medium at 37°C for 24 h to obtain the leachate. The extract was collected, centrifuged to remove particulates if necessary, and sterilized by filtration (0.22 μm).

To prepare NBP@Gel extract, the NBP@Gel formulation was incubated in complete culture medium at 37°C for 24 h to obtain the leachate. The extract was collected, centrifuged to remove particulates if necessary, and sterilized by filtration (0.22 μm). The total amount of NBP incorporated into NBP@Gel was quantified by HPLC and normalized to match the free NBP dose used in cell experiments.

### 2.26 CCK-8 assay

N2a cells were seeded in 96-well plates and allowed to adhere overnight. For concentration evaluation, cells were treated with free NBP at 0, 50, 100, 200, and 300 μM for 24 h. For assessment of treatment effects, cells were treated with Gel extract, free NBP (200 μM), or NBP@Gel extract (The total amount of NBP incorporated into NBP@Gel was normalized to the free NBP dose of 200 μM). Cell viability was then measured using the CCK-8 assay.

### 2.27 Cell treatments

For direct in vitro experiments, BV2 or N2a cells were seeded in culture plates and allowed to adhere overnight. BV2 cells were assigned to the following groups according to the specific experimental design: (i) Control; (ii) LPS; (iii) LPS + NBP; (iv) LPS + NBP + DIF-3; (v) LPS + NBP + ML385; and (vi) LPS + NBP@Gel. For treatment, LPS (1 μg/mL) was added simultaneously with the interventions, including NBP, DIF-3, ML385, or NBP@Gel, according to the corresponding experimental groups, and cells were cultured for 24 h. For N2a treatment, conditioned medium collected from LPS-stimulated BV2 cells was used to establish an inflammatory injury condition, and N2a cells were simultaneously treated with the interventions, including NBP, DIF-3, ML385, or NBP@Gel, according to the corresponding experimental groups, for 24 h. After treatment, BV2 or N2a cells were collected for downstream assays.

### 2.28 Transwell co-culture of BV2 and N2a cells or primary neurons

For indirect co-culture, N2a cells or primary neurons were seeded in the lower chamber of 24-well plates and used for co-culture when they reached approximately 60–70% confluence. BV2 cells were seeded in 0.4-μm pore Transwell inserts (Corning, 3413). Before co-culture, BV2 cells were assigned to the following groups: (i) Control; (ii) LPS; (iii) LPS + NBP; and (iv) LPS + NBP@Gel. BV2 cells were treated simultaneously with LPS (1 μg/mL) and the corresponding intervention, including NBP or NBP@Gel for 24 h. After treatment, BV2 cells were washed twice with PBS, changed to normal medium, and transferred above N2a cells or primary neurons for an additional 24 h of indirect co-culture. After co-culture, N2a cells or primary neurons in the lower chamber were collected for downstream analyses.

### 2.29 Annexin V-FITC/PI apoptosis assay

Apoptosis was quantified by Annexin V–FITC/PI staining followed by flow cytometry. Cells were collected by trypsinization using 0.25% trypsin (EDTA-free; Gibco, 15050065) for 2 min at 37°C, neutralized with DMEM containing 10% FBS (Gibco, 11965118), and centrifuged at 1000 rpm for 5 min. Cell pellets were washed twice with PBS and resuspended. For staining, 100 μL cell suspension was transferred to FACS tubes (BD, 352052), mixed with 5 μL Annexin V–FITC and 5 μL PI from the apoptosis kit (Solarbio, CA1020), and incubated for 15 min at room temperature in the dark. PBS (400 μL) was then added prior to acquisition. Samples were analyzed on a BD FACSCalibur flow cytometer, and total apoptosis was calculated as the sum of early and late apoptotic populations using FlowJo.

### 2.30 Live/dead staining

N2a neurons were treated for 24 h with Gel extract, NBP, or NBP@Gel extract, as indicated for the four conditions (Control, Gel, NBP, and NBP@Gel), and then subjected to Live/Dead viability staining. N2a cells on coverslips were gently rinsed twice with pre-warmed PBS. Cells were then incubated with Live/Dead working solution containing Calcein-AM (to label live cells) and propidium iodide (PI; to label dead cells), prepared according to the manufacturer's instructions (Solarbio, CA1630), for 15–30 min at 37°C in the dark. After staining, cells were washed twice with PBS and immediately imaged using a fluorescence microscope under identical exposure settings across groups. Cell viability was quantified by counting Calcein-positive (live) and PI-positive (dead) cells in randomly selected fields, and the percentage of live cells was calculated for each condition.

### 2.31 Statistical analysis

Statistical analyses were performed using Prism software version 7.0 (GraphPad Software, San Diego, CA, USA). All data were expressed as the mean ± standard error of mean (SEM). Comparison of two groups was performed using Student's t-test. Multiple comparisons were done using the one-way or two-way ANOVA followed by Tukey's post hoc test. All tests were considered statistically significant at p < 0.05.

## 3 Results

### 3.1 Iron metabolism and ferroptosis represent key pathological features of TBI

**Fig. 1:**
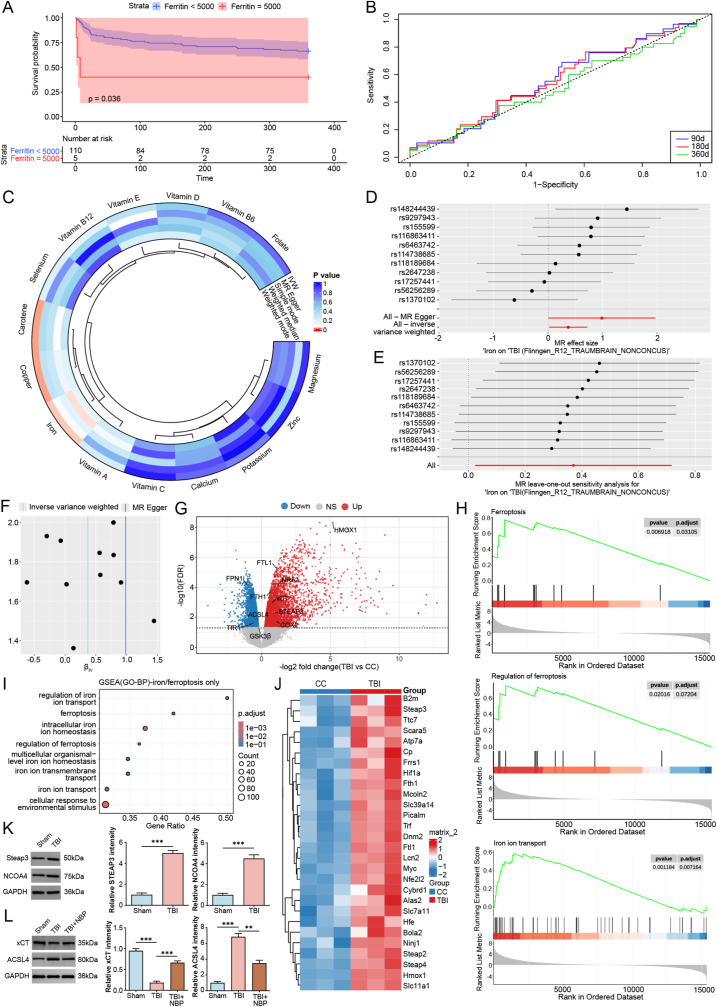
**Clinical, genetic, and transcriptomic evidence implicating iron metabolism and ferroptosis as key factors in TBI.** (A) Kaplan-Meier survival curves for TBI patients stratified by baseline ferritin, showing significantly lower survival rate the high-ferritin group. (B) Time-dependent ROC analysis indicating ferritin's prognostic value for TBI outcomes over time. (C) Circos plot summarizing Mendelian randomization (MR) associations between circulating micronutrients and TBI risk (color indicates P value). (D) Forest plot of SNP-specific and pooled MR estimates for genetically proxied serum iron on TBI risk. (E) Leave-one-out sensitivity analysis for the MR results. (F) MR scatter plot showing SNP-specific effects with IVW and MR-Egger fits. (G) Volcano plot of differential gene expression between TBI and control (CC) groups (day 3). (H) GSEA enrichment plots for ferroptosis, regulation of ferroptosis, and iron ion transport-related gene sets. (I) Bubble plot summarizing selected GO biological processes related to iron metabolism/ferroptosis. (J) Heatmap of leading-edge genes from iron/ferroptosis-related gene sets comparing CC and TBI samples. (K) Western blot analysis and quantification of STEAP3 and NCOA4 (Sham and TBI groups).(L)Western blot analysis and quantification of xCT and ACSL4 (Sham,TBI,and TBI + NBP groups). All quantitative data are presented as mean ± SEM (K-L, n = 5 mice per group). Two-group comparisons used Student's t-test; multiple-group comparisons used one-way ANOVA with Tukey's post hoc test. ∗∗P < 0.01, ∗∗∗P < 0.001. (For interpretation of the references to color in this figure legend, the reader is referred to the Web version of this article.)

To evaluate whether iron metabolism is related with outcomes after TBI, we first examined clinical survival data. Patients were stratified by baseline ferritin, and Kaplan-Meier analysis showed a significantly lower survival rate in the high-ferritin group (Fig. 1A).Consistently, time-dependent ROC curves indicated that ferritin provides prognostic value for TBI outcomes across follow-up (Fig. 1B), supporting a link between iron metabolism and adverse prognosis. Building on this association, we next applied MR to test the potential causal contribution of circulating micronutrients to TBI risk. Among these micronutrients, the Circos plot indicated iron levels were significantly related with TBI risk (Fig. 1C). Moreover, SNP-specific and pooled MR estimates further supported a positive association between genetically proxied serum iron and TBI risk (Fig. 1D). Sensitivity analysis using leave-one-out testing showed that the iron-TBI estimate remained stable after removing individual instruments one at a time (Fig. 1E). This indicates that the effect was not driven by any single SNP. MR scatter plots showed directionality of SNP effects and similar fitted lines for IVW and MR-Egger models (Fig. 1F), supporting the robustness of the MR inference. These results indicated iron metabolism is important for TBI progression. Iron metabolism is strongly related with ferroptosis, and to explore whether iron metabolism and ferroptosis are altered after TBI, we tested whether brain transcriptomic programs reflect iron-related biology after TBI by analyzing a mouse TBI dataset (GSE79441).Differential expression analysis of the acute-phase (day 3) mouse TBI dataset (GSE79441) showed marked changes between TBI and control (CC) samples. These included changes in iron-handling and stress-response genes, such as Steap3, Fth1, Ftl1, Nrf2, and Cox2 (Fig. 1G). Gene set enrichment analyses showed positive enrichment of ferroptosis, regulation of ferroptosis, and iron ion transport-related gene sets (Fig. 1H). A focused bubble plot further summarized selected GO biological processes linked to iron metabolism and ferroptosis (Fig. 1I). A heatmap of leading-edge genes from iron/ferroptosis-related gene sets revealed a coordinated transcriptional shift distinguishing TBI from control (CC) samples, including altered expression of key iron-handling and ferroptosis regulators such as Steap3, Fth1, Ftl1 (Fig. 1J). Then, we validated whether ferroptosis is increased after TBI using a mouse TBI model. Western blotting and quantification showed that ferroptosis-related proteins STEAP3 and NCOA4 which connect iron metabolism and ferroptosis were increased in the TBI group compared with Sham (day 3) (Fig. 1K). The western blot results of another ferroptosis-related protein ACSL4 exhibit the same trend,the expression of it was elevated after TBI and decreased after NBP treatment, whereas xCT was reduced after TBI and increased after NBP treatment (Fig. 1L). Collectively, these results implicated ferroptosis as a key component of TBI pathophysiology, is a promising therapeutic target in acute phase of TBI.

### 3.2 Network pharmacology identifies GSK-3β as a key target of NBP in TBI

**Fig. 2:**
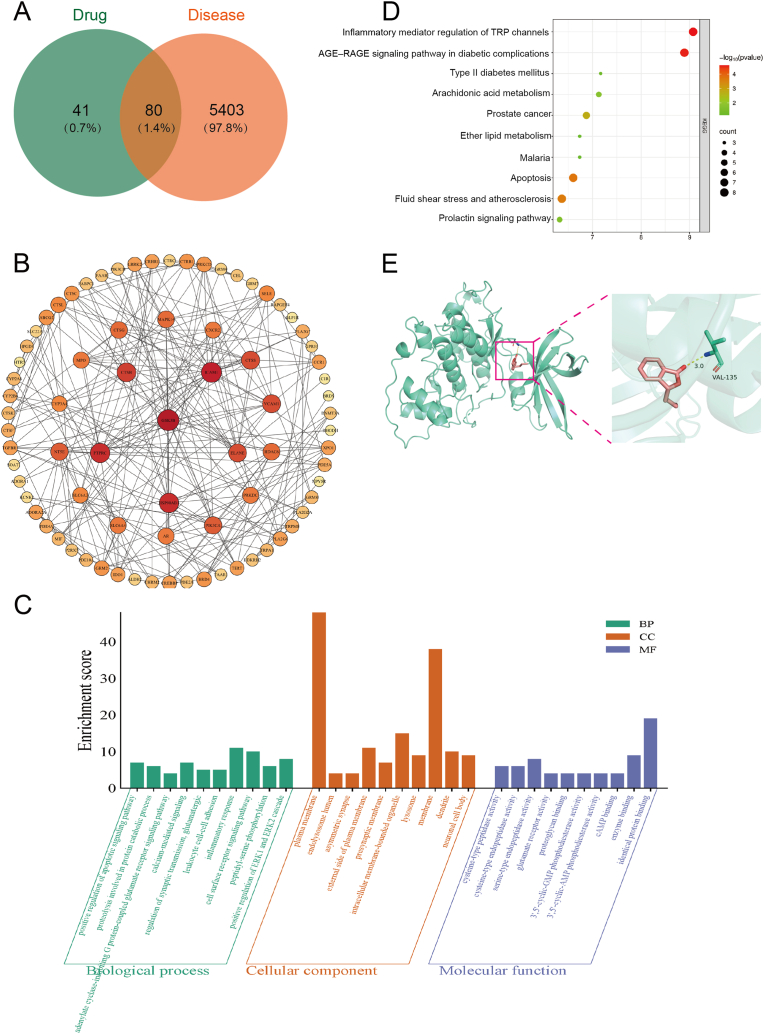
**Target prediction and molecular interaction analysis of NBP in TBI based on network pharmacology.** (A) Venn diagram of shared targets between NBP-related targets and TBI-associated therapeutic targets, identifying 80 common targets. (B) Protein–protein interaction (PPI) network of the common targets, where nodes represent proteins and edges indicate known or predicted protein–protein associations, highlighting potential hub targets within the network. (C) Gene Ontology (GO) enrichment analysis of the common targets. Enriched terms (FDR <0.05) are summarized across Biological Process (BP), Cellular Component (CC), and Molecular Function (MF); bar height indicates enrichment significance and colors denote GO categories (BP, CC, MF). (D) KEGG pathway enrichment bubble chart of the top enriched pathways. The X-axis shows gene ratio, the Y-axis lists pathway names; bubble size reflects the number of genes mapped to each pathway, and color intensity corresponds to enrichment significance. (E) Molecular docking of NBP with GSK-3β, showing the predicted binding pose within the active pocket and a zoomed-in view of key interactions. (For interpretation of the references to color in this figure legend, the reader is referred to the Web version of this article.)

Network pharmacology analysis was performed to explore the protective mechanism of NBP in TBI. We intersected NBP-related targets with TBI therapeutic targets and identified 80 common genes. These genes were considered potential therapeutic targets of NBP for further investigation (Fig. 2A). A protein–protein interaction (PPI) network constructed from these common genes revealed a highly connected module centered on GSK-3β. This suggests that kinase-centered signaling may be a principal node through which NBP exerts activity (Fig. 2B). To further investigate the biological mechanisms, we performed Gene Ontology enrichment analysis. The top biological process terms included inflammatory response and cell surface receptor signaling pathway. This indicates that NBP might regulate immune responses and receptor-mediated signaling in the context of TBI (Fig. 2C). For cellular component, the analysis highlighted terms such as plasma membrane, membrane, and intracellular membrane-bounded organelle. This suggests that NBP might interact with cellular structures involved in membrane dynamics and signal transduction. The molecular function terms, such as identical protein binding, enzyme binding, and cAMP binding, point to potential interactions between NBP and various molecular components involved in signaling and cellular regulation (Fig. 2C). The KEGG pathway enrichment analysis revealed that several pathways related to inflammatory response, stress response, and cell signaling were significantly enriched. This indicates that NBP might regulate key pathways involved in oxidative stress and inflammatory responses (Fig. 2D). Molecular docking was performed to explore the binding interaction between NBP and GSK-3β. Docking analysis showed that NBP binds effectively to GSK-3β. It occupies a hydrophobic pocket near Val135, forming strong hydrophobic interactions and a hydrogen bond with Val135 at 3.02 Å (Fig. 2E). This stable binding indicates that NBP can modulate GSK-3β activity, affecting downstream inflammatory and oxidative stress pathways in TBI recovery. These interactions produce a moderate binding affinity of −6.3 kcal/mol, explaining how NBP binds and modulates GSK-3β function to support its neuroprotective effects in TBI.

### 3.3 NBP attenuates LPS-induced ferroptosis through the GSK-3β-Fyn-Nrf2 pathway

**Fig. 3:**
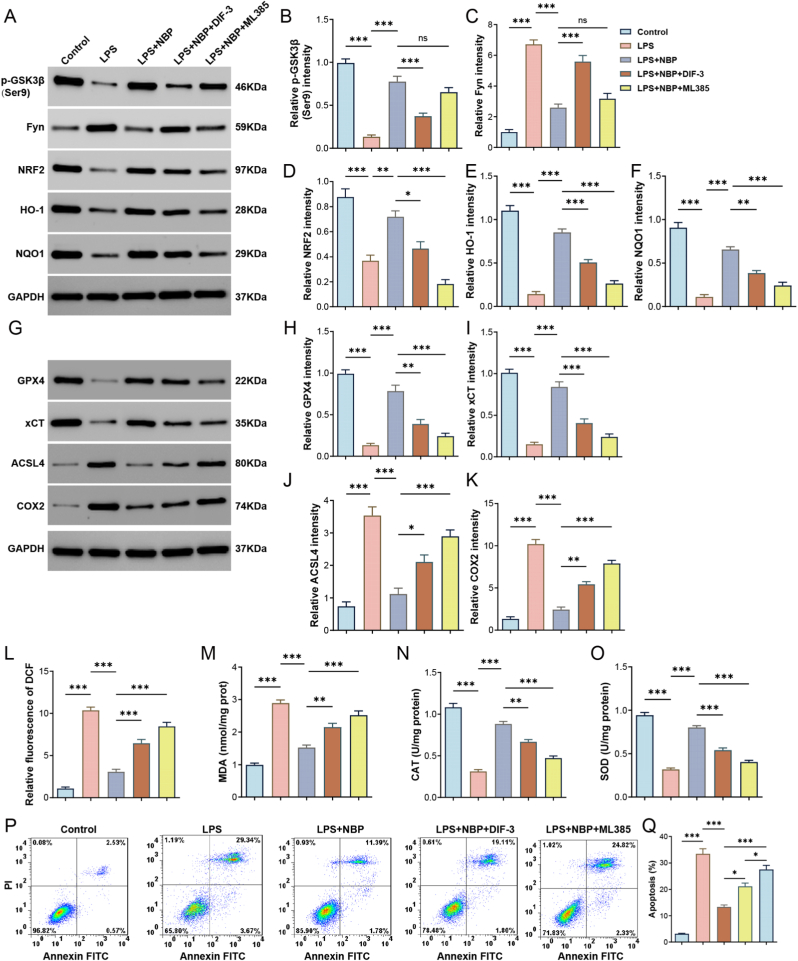
NBP inhibits ferroptosis by suppressing GSK-3β and activating the Nrf2 axis. (A) Representative western blot bands of p-GSK-3β(Ser9), Fyn, NRF2, HO-1, and NQO1. (B–F) Densitometric quantification of p-GSK-3β (Ser9) (B), Fyn (C), NRF2 (D), HO-1 (E), and NQO1 (F) in N2a cells. (G) Representative western blot bands of ferroptosis-related proteins GPX4, xCT,ACSL4, and COX-2. (H–K) Densitometric quantification of GPX4 (H), xCT (I), ACSL4 (J), and COX-2 (K) in N2a cells. (L) Intracellular ROS levels measured using the DCFH-DA probe and expressed as relative DCF fluorescence. (M) MDA level. (N) CAT activity. (O) SOD activity in N2a cells. (P) Representative flow cytometry plots of Annexin V–FITC/PI staining in each group. (Q) Quantification of apoptosis rate from Annexin V–FITC/PI analysis in N2a cells. All quantitative data are presented as mean ± SEM (n = 3 independent experiments). Statistical significance was assessed by one-way ANOVA followed by Tukey's post hoc test.ns, not significant; ∗P < 0.05, ∗∗P < 0.01, ∗∗∗P < 0.001.

To determine whether protective effects of NBP are associated with GSK-3β and NRF2 signaling, we introduced DIF-3 (GSK-3β activator) and ML385 (NRF2 inhibitor). Based on the results of the cytotoxicity assessment of NBP in N2a cells, 200 μM was selected as the maximum working concentration for subsequent in vitro experiments(Fig. S4A). The results of western blot revealed that LPS considerably altered GSK-3β-Fyn-Nrf2 pathway proteins compared to controls, while NBP dramatically corrected these LPS-induced changes. Notably, when DIF-3 was added, all five readouts (GSK-3β, Fyn, Nrf2, HO-1, and NQO1) differed significantly compared with the LPS + NBP group (Fig. 3A–F). By comparison, the addition of ML385 was related with significant changes limited to Nrf2, HO-1, and NQO1, while GSK-3β and Fyn remained unchanged relative to the LPS + NBP group (Fig. 3A–F), further supporting that NBP modulates the GSK-3β–Fyn–Nrf2 pathway. HO-1 and NQO1 are downstream targets of Nrf2 and have been implicated in the regulation of ferroptosis, suggesting that NBP may suppress ferroptosis partly through the GSK-3β-Fyn-Nrf2 axis. To investigate whether NBP inhibits ferroptosis via GSK-3β-Fyn-Nrf2 pathway following LPS-induced injury, we detected ferroptosis-related proteins using WB. The results showed that LPS decreased GPX4 and xCT and increased ACSL4 and COX-2 relative to controls. NBP significantly reversed these changes. Treatment with DIF-3 or ML385 partially attenuated the changes observed in the NBP-treated group, with GPX4/xCT decreasing and ACSL4/COX-2 increasing relative to the LPS + NBP group (Fig. 3G–K). Since oxidative stress is closely related with ferroptosis, we assessed oxidative stress levels. Oxidative-stress readouts showed consistent group differences. Relative DCF fluorescence (ROS level) (Fig. 3L) and MDA level (Fig. 3M) were significantly increased by LPS and reduced by NBP. However, DIF-3 or ML385 co-treatment partially reversed the NBP-mediated reductions. In parallel, CAT activity and SOD activity were decreased in the LPS group and restored by NBP, whereas DIF-3 or ML385 attenuated these restorative effects (Fig. 3N–O). In addition, DIF-3 or ML385 partially reversed the effects of NBP on apoptosis and inflammatory readouts (Fig. 3A–B and S1).

### 3.4 Fabrication and physicochemical characterization of NBP-loaded injectable hydrogels

**Fig. 4:**
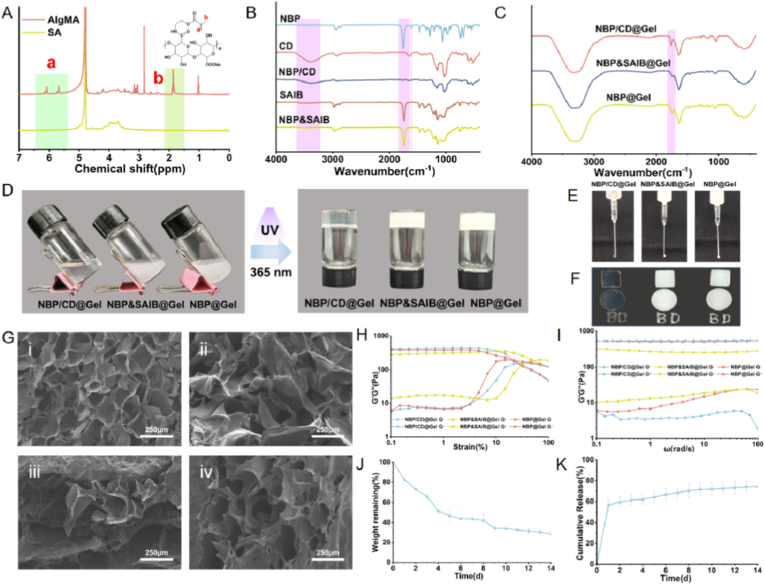
**Preparation and characterization of NBP-loaded injectable hydrogels.** (A) ^1H NMR spectra of sodium alginate and methacrylated alginate. (B) FT-IR spectra of NBP, SBE-β-CD, NBP/CD, SAIB, and SAIB&NBP. (C) FT-IR spectra of NBP-loaded hydrogels (NBP/CD@Gel, NBP&SAIB@Gel, and the composite hydrogel NBP/CD + NBP&SAIB@Gel, hereafter referred to as NBP@Gel for simplicity). (D) Photographs of hydrogel precursor solutions before and after UV irradiation (365 nm). (E) Injectability of hydrogel precursor solutions through a syringe needle. (F) Shape-molding ability of the formed hydrogels. (G) SEM images of hydrogel microstructures (i Blank@Gel; ii NBP/CD@Gel; iii NBP @Gel; iv NBP@Gel). Scale bar = 25 μm. (H) Strain-dependent rheological properties of the hydrogels. (I) Frequency-dependent rheological properties of the hydrogels. (J) In vitro degradation profile of NBP@Gel in PBS (pH 7.4). (K) In vitro cumulative release of NBP from NBP@Gel over 14 days.All quantitative data are presented as mean ± SEM (H-K, n = 3 independent samples).

Based on our findings and previous studies, NBP can inhibit ferroptosis in acute phase and regulate microglial polarization during subacute and chronic phase. Thus, we developed a hydrogel that enables both rapid and sustained release of NBP after TBI. The chemical structure of methacrylated alginate (AlgMA) was unequivocally confirmed by ^1^H NMR spectroscopy. As depicted in Fig. 4A, the characteristic proton signals of the alginate backbone were observed in the range of δ4.20–3.40 ppm. Following grafting with 2-aminoethyl methacrylate (AEME), distinctive new resonances emerged at δ1.86, 5.67, and 6.08 ppm, which are indicative of the successful conjugation of methacryloyl (MA) groups to the amine functionalities of sodium alginate (SA). Furthermore, the signals at δ5.58 and 6.05 ppm can be assigned to the vinyl protons (-C=CH_2_) of the methacrylate moiety, providing additional evidence for the successful synthesis of AlgMA. The infrared spectra of NBP, SBE-β-CD, and SAIB are shown in Fig. 4B. The active pharmaceutical ingredient NBP exhibited a carbonyl (C=O) stretching vibration absorption peak at 1754 cm and a C=C stretching vibration peak at 1614 cm. SBE-β-CD displayed a characteristic hydroxyl (-OH) absorption peak at 3402 cm. After complexation of NBP with SBE-β-CD, the -OH absorption peak remained present in the NBP@CD spectrum, while the characteristic peak at 1754 cm was still weakly detectable. This is attributed to the partial embedding of NBP molecules into the SBE-β-CD cavity, leaving some C=O groups exposed on the exterior, which confirms the successful formation of the inclusion complex between SBE-β-CD and NBP. Upon uniform mixing of SAIB with NBP, the -OH absorption peaks of both components overlapped, and the characteristic peak of NBP at 1614 cm, remained visible without the emergence of any new absorption bands. This indicates that no chemical reaction occurred between SAIB and NBP upon blending. Three hybrid hydrogel systems were formulated by incorporating NBP@CD and a physical mixture of NBP & SAIB into a hydrogel precursor solution, respectively. As illustrated in the Fourier transform infrared (FT-IR) spectra (Fig. 4C), characteristic absorption peaks attributable to the carbonyl group (C=O) of NBP were detected at approximately 1758 cm^−1^ in all three hybrid hydrogels, confirming the successful integration of the drug within the polymeric networks. Fig. 4D demonstrates that the precursor solutions of all three hydrogels exhibited a slightly viscous liquid state at room temperature. When exposed to 365 nm ultraviolet light, these solutions underwent efficient cross-linking, transitioning into solid hydrogels. The injectability of the system was qualitatively assessed by extruding the pre-gel solutions seamlessly through a 22-gauge syringe needle (Fig. 4E). The exceptional shape adaptability of hydrogel was demonstrated by their ability to be molded into a variety of architectures using silicone molds, as illustrated in Fig. 4F. Its exceptional injectability and ease of handling were further demonstrated by the fact that the letters "BD" were written directly with a syringe filled with the solid hydrogel. The morphological features of the four hydrogels after reaching equilibrium swelling in deionized water at 25°C were examined by scanning electron microscopy (SEM). As shown in Fig. 4G, the Blank@Gel (i), NBP/CD@Gel (ii), NBP&SAIB@Gel (iii), and NBP@Gel (iv) all exhibited a porous and interconnected architecture. Pore sizes were distributed in the range of 71–121 μm. As shown in Fig. 4H and I, the hydrogels had storage moduli (G′) ranging from 250 to 550 Pa. In the strain sweep test (Fig. 4H), both G′ and the loss modulus (G″) were generally constant at low strain, but decreased at higher strain, indicating disruption of the network structure. In the frequency sweep test (Fig. 4I), G′ and G″ showed very little reliance on frequency in the range that was examined. This suggests that the material has stable viscoelastic behavior that is compatible with soft brain tissue.We evaluated the degradation profile of NBP@Gel in PBS (pH 7.4) at 37°C for 14 days (Fig. 4J). After 14 days, the hydrogel preserved 27.7 ± 2.7% of its initial mass, demonstrating good structural stability under physiological settings and a comparatively moderate rate of deterioration. The in vitro drug release behavior of NBP@Gel is shown in Fig. 4K. An initial burst release was observed on day 1 (56.62 ± 5.40%), which was followed by a continuous release phase over the next 13 days, culminating in a cumulative release of 74.62 ± 6.28% by day 14, suggesting that NBP@Gel can serve as an effective platform for sustained NBP delivery. In vivo fluorescence imaging revealed that the free formulation signal was undetectable by day 7, whereas the hydrogel formulation exhibited persistent fluorescence at the lesion region with signal retention still clearly detectable at 14 days post-implantation (Fig. S5). These findings indicate that the hydrogel system maintains local retention for more than 14 days, thereby supporting sustained drug delivery during the chronic phase of TBI.

### 3.5 NBP@Gel promotes long-term neural function recovery and neuronal repair in a mouse TBI model

**Fig. 5:**
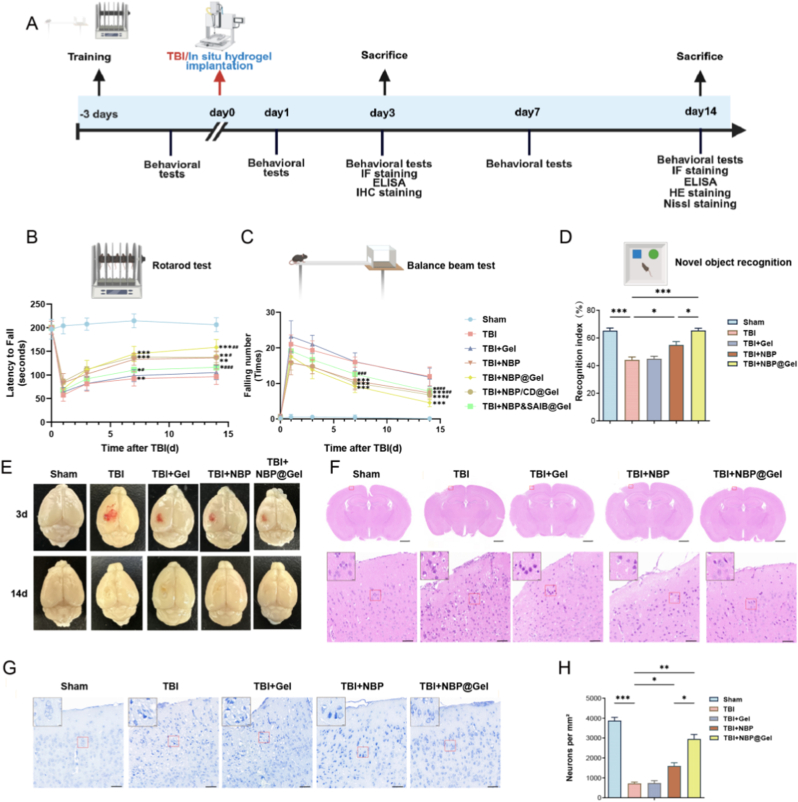
**In situ NBP@Gel implantation improves functional recovery and neuronal repair after TBI in vivo**. (A) Schematic timeline of the animal experiment. (B) Rotarod performance shown as latency to fall at different time points after TBI in the Sham, TBI, TBI + Gel, TBI + NBP, TBI + NBP@Gel, TBI + NBP/CD@Gel and TBI + NBP&SAIB@Gel groups. (C) Beam-walking assessment shown as the number of falls at different time points after TBI in each group. (D) Novel object recognition assessment on day 14 after TBI. (E) Representative gross images of brains from each group on days 3 and 14 after TBI. (F) Representative H&E-stained coronal brain sections (upper) and magnified views of the pericontusional region (lower) from each group at day 14. Insets show enlarged views of the boxed areas. Scale bars: upper, 1000 μm; lower, 50 μm; insets, 5 μm. (G) Representative Nissl staining images of the pericontusional cortex from each group at day 14. Insets show enlarged views of the boxed areas. Scale bars: 50 μm; insets, 5 μm. (H) Quantification of surviving neurons in the pericontusional cortex for each group. All quantitative data are presented as mean ± SEM (B-D, n = 9; H, n = 5 mice per group). Statistical significance was assessed by one-way ANOVA followed by Tukey's post hoc test. In B and C, ∗ indicates comparison with the TBI group and # indicates comparison with the TBI + NBP@Gel group. ns, not significant; ∗P < 0.05, ∗∗P < 0.01, ∗∗∗P < 0.001; #P < 0.05, ##P < 0.01, ###P < 0.001.

We assessed the therapeutic efficacy of in situ NBP@Gel implantation in a mouse TBI model. The experimental timeline is shown in Fig. 5A. We first evaluated motor coordination and balance using the rota-rod test. TBI significantly shortened the latency to fall. Compared with the TBI group, animals treated with NBP@Gel showed a clear improvement in latency to fall over time (Fig. 5B). While NBP, NBP/CD@Gel, and NBP&SAIB@Gel all improved rotarod performance to some extent, the NBP@Gel group exhibited the best long-term outcomes, particularly at later time points (Fig. 5B). In the falling number test, TBI markedly increased falling numbers, whereas NBP@Gel substantially reduced the falling number during the follow-up period (Fig. 5C). Based on these results, the composite NBP@Gel formulation was identified as the lead group among all hydrogel-based formulations, exhibiting superior behavioral recovery compared to both the immediate-release (NBP/CD@Gel) and sustained-release (NBP&SAIB@Gel) gels alone. For all subsequent experiments, five groups were maintained: Sham, TBI, TBI + Gel, TBI + NBP, and TBI + NBP@Gel. In the NOR test, TBI markedly reduced the NOR index, whereas NBP@Gel substantially improved cognitive performance at day 14 (Fig. 5D). Although NBP improved recognition to the novel object, NBP@Gel achieved a more pronounced improvement, indicating a sustained benefit on post-TBI cognitive function. Brain images revealed prominent injury signs in the TBI group at day 3 after TBI. Both the NBP and NBP@Gel treatment groups exhibited a reduced injury area compared with the TBI and TBI + Gel groups. On day 14 after TBI, the severity of the injury was diminished across all groups. In contrast to the TBI and TBI + Gel groups, the NBP treatment group and the NBP@Gel group displayed a decreased injury area; of all the groups, the NBP@Gel group displayed the smallest injury area(Fig. 5E). On day 14 post-TBI, H&E staining was performed to observe the pericontusional cortex. The TBI group displayed pronounced structural disruption and neuron loss in the peri-injury region. This disruption was partially alleviated by NBP and was further attenuated in the NBP@Gel group, which exhibited the most maintained cortical architecture and neuronal integrity among the treatment groups (Fig. 5F). Nissl staining consistently demonstrated enhanced neuronal preservation in the NBP treatment group, with the NBP@Gel group displaying the most significant preservation of neuronal integrity among the treatment groups (Fig. 5G). Quantification of surviving neurons supported this protective effect, revealing considerably increased neuronal density in the NBP@Gel group (Fig. 5H). Overall, these findings show that in situ NBP@Gel implantation enhances long-term histological healing and neuronal preservation.

### 3.6 NBP@Gel mitigates oxidative damage and ferroptosis-related changes after TBI

**Fig. 6:**
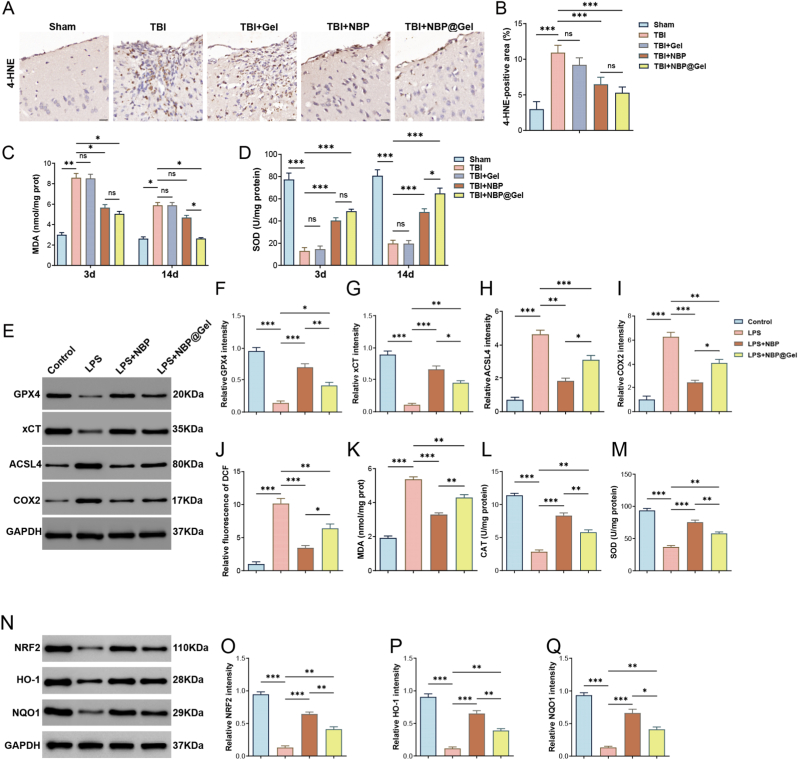
**NBP@Gel alleviates oxidative stress and ferroptosis in vivo and in vitro.** (A) Representative immunohistochemical staining of 4-HNE in brain sections from Sham, TBI, TBI + Gel, TBI + NBP, and TBI + NBP@Gel group at day 3. Scale bar = 20 μm. (B) Quantification of 4-HNE-positive area. (C) MDA levels in brain tissue at day 3 and day 14 post-TBI. (D) SOD activity in brain tissues at day 3 and day 14 post-TBI. (E) Representative Western blot bands of GPX4, xCT, ACSL4, and COX2 in N2a cells. (F–I) Densitometric analysis of GPX4, xCT, ACSL4, and COX2 protein levels in N2a cells, respectively. (J) Intracellular ROS level in N2a cells measured by DCF fluorescence. (K–M) Oxidative stress–related indices in N2a cells, including MDA level, CAT activity, and SOD activity. (N) Representative Western blot bands of NRF2, HO-1, and NQO1 in N2a cells. (O–Q) Densitometric analysis of NRF2, HO-1, and NQO1 protein levels in N2a cells. All quantitative data are presented as mean ± SEM (B-D, n = 5 mice per group; F-M and O-Q, n = 3 independent experiments). Statistical significance was assessed by one-way ANOVA followed by Tukey's post hoc test. ns, not significant; ∗P < 0.05, ∗∗P < 0.01, ∗∗∗P < 0.001.

To evaluate oxidative damage and ferroptosis-related changes after TBI, we assessed lipid peroxidation and antioxidant indices in brain tissue (Fig. 6A–D). On day 3 post-TBI, 4-HNE immunostaining was markedly increased in the TBI group compared with Sham, indicating enhanced lipid peroxidation (Fig. 6A). Quantitative analysis confirmed a significant elevation of the 4-HNE positive area in the TBI group. NBP and NBP@Gel treatment significantly reduced 4-HNE expression (Fig. 6B). Consistently, MDA and ROS levels in brain tissue were elevated after TBI at both day 3 and day 14 (Fig. 6C and S2). Both NBP and NBP@Gel significantly decreased MDA and ROS compared with the TBI group at these time points (Fig. 6C and S2). At day 14, the NBP@Gel group showed a greater reduction in MDA and ROS than the NBP group, suggesting improved long-term control of lipid peroxidation (Fig. 6C and S2). In parallel, SOD and CAT activity was significantly suppressed in the TBI group at both time points. Treatment with NBP or NBP@Gel significantly restored SOD and CAT activity, with NBP@Gel exhibiting a larger improvement at day 14(Fig. 6D and S2). This indicates a more sustained enhancement of antioxidant capacity in vivo. In vitro, ferroptosis-related and oxidative stress–related markers were examined in N2a cells (Fig. 6E–Q). LPS stimulation induced changes consistent with ferroptosis-related oxidative injury. These changes included decreased expression of GPX4 and xCT and increased levels of ACSL4 and COX2 (Fig. 6E–I). Both LPS + NBP and LPS + NBP@Gel significantly counteracted these LPS-induced protein changes compared to LPS (Fig. 6E–I). NBP showed a stronger overall effect than NBP@Gel in restoring these protein levels quantitatively (Fig. 6E–I). These molecular changes were accompanied by functional oxidative stress indices. LPS markedly increased intracellular ROS and MDA, while reducing CAT and SOD activities (Fig. 6J–M). Both NBP and NBP@Gel significantly lowered ROS and MDA and increased CAT and SOD relative to LPS. NBP showed a stronger overall effect than NBP@Gel across these assays (Fig. 6J–M). Similarly, both NBP and NBP@Gel attenuated LPS-induced apoptosis in N2a cells, with NBP exhibiting a relatively stronger effect than NBP@Gel (Fig. S3).Finally, LPS suppressed the NRF2 antioxidant pathway, decreasing NRF2, HO-1, and NQO1 expression (Fig. 6N–Q). Both NBP and NBP@Gel treatments significantly restored NRF2 signaling compared with LPS, and NBP resulted in higher NRF2/HO-1/NQO1 levels than NBP@Gel in the quantified results (Fig. 6N–Q). Overall, the in vivo data indicate that both NBP and NBP@Gel attenuate lipid peroxidation and improve antioxidant defense after TBI, with NBP@Gel showing a more sustained benefit at later stages. Consistently, in N2a cells, both NBP and NBP@Gel alleviated ferroptosis- and oxidative stress-related alterations, whereas NBP tended to produce a stronger overall improvement.These findings suggest that NBP has a protective effect against ferroptosis after TBI.

### 3.7 NBP@Gel promotes a sustained anti-inflammatory microglial phenotype shift after TBI

**Fig. 7:**
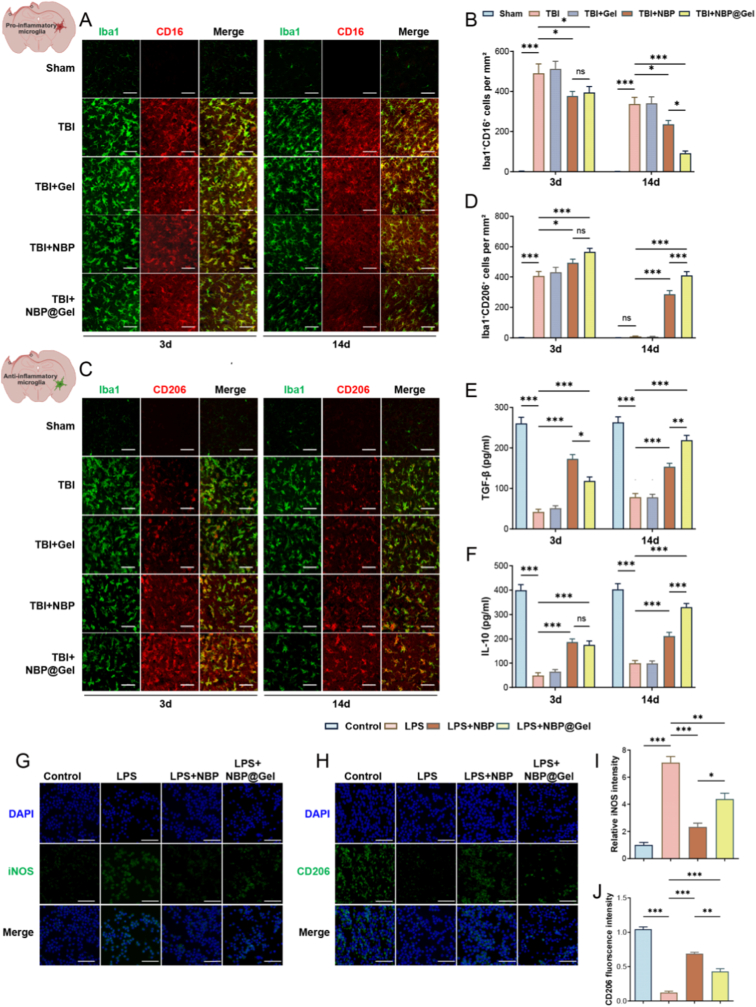
**NBP@Gel modulates microglial polarization and inflammatory cytokine in vivo and in vitro.** (A) Representative immunofluorescence images of pro-inflammatory microglia in peri-injury brain regions, stained for Iba1 (green) and CD16 (red), with merged images shown in the Sham, TBI, TBI + Gel, TBI + NBP, and TBI + NBP@Gel groups at day 3 and day 14. Scale bars: 50 μm. (B) Quantification of Iba1^+^CD16^+^ cells at day 3 and day 14. (C) Representative immunofluorescence images of anti-inflammatory microglia, stained for Iba1 (green) and CD206 (red), with merged images shown in the Sham, TBI, TBI + Gel, TBI + NBP, and TBI + NBP@Gel groups at day 3 and day 14. Scale bars: 50 μm. (D) Quantification of Iba1^+^CD206^+^ cells at day 3 and day 14. (E) TGF-β levels measured by ELISA at day 3 and day 14 in each group. (F) IL-10 levels measured by ELISA at day 3 and day 14 in each group. (G) Representative immunofluorescence staining images of iNOS in BV2 microglias across groups. Nuclei were counterstained with DAPI. Scale bars:100 μm. (H) Representative immunofluorescence staining images of CD206 in BV2 microglias across groups. Nuclei were counterstained with DAPI. Scale bars:100 μm. (I) Quantitative analysis of iNOS fluorescence intensity in BV2 microglias across groups. (J) Quantitative analysis of CD206 fluorescence intensity in BV2 microglias across groups. All quantitative data are presented as mean ± SEM (B, D-F, n = 5 mice per group; I-J, n = 3 independent experiments). Statistical significance was assessed by one-way ANOVA followed by Tukey's post hoc test. ns, not significant; ∗P < 0.05, ∗∗P < 0.01, ∗∗∗P < 0.001. (For interpretation of the references to color in this figure legend, the reader is referred to the Web version of this article.)

We subsequently conducted immunofluorescence staining of anti-inflammatory and pro-inflammatory microglia to investigate the modulatory effects of NBP@Gel on microglial responses after TBI. The results showed that the TBI group displayed a marked increase in CD16 expression and a higher proportion of Iba1^+^CD16^+^ cells compared with Sham, indicating an enhanced pro-inflammatory microglial response. Quantification further confirmed a significant rise in Iba1^+^CD16^+^ cell density after TBI (Fig. 7A and B). At day 3, both the NBP and NBP@Gel groups showed reduced Iba1^+^CD16^+^ cell density compared with the TBI and TBI+Gel groups, with no significant difference between NBP and NBP@Gel (Fig. 7A and B). By day 14, the pro-inflammatory microglial population declined across groups, but the reduction was most pronounced in the NBP@Gel group, which exhibited significantly fewer Iba1^+^CD16^+^ cells than both the TBI and NBP groups, supporting a stronger long-term suppression of the pro-inflammatory phenotype (Fig. 7A and B). We further assessed the anti-inflammatory microglial marker CD206. Representative Iba1^+^CD206^+^ double-immunofluorescence in the peri-injured cortex showed increased CD206 signal and a higher density of Iba1^+^CD206^+^ cells in NBP and NBP@Gel treatment groups relative to TBI (Fig. 7C). Quantification confirmed that, at day 3, both NBP and NBP@Gel increased the density of Iba1^+^CD206^+^ cells compared with TBI+Gel (Fig. 7D). By day 14, CD206-positive microglia declined in the TBI and TBI + Gel groups, whereas both NBP and NBP@Gel maintained significantly higher Iba1^+^CD206^+^ cell density, with NBP@Gel showing a greater effect (Fig. 7D). In parallel, ELISA showed that both TGF-β and IL-10 were markedly reduced after TBI at day 3 and day 14 (Fig. 7E and F). At day 3, both NBP and NBP@Gel increased TGF-β and IL-10 relative to the TBI and TBI + Gel groups, indicating an early immunomodulatory effect (Fig. 7E and F). At day 3, IL-10 levels were comparable between the NBP and NBP@Gel groups, and TGF-β was even higher in the NBP group. By day 14, both TGF-β and IL-10 were higher in the NBP@Gel group than in the NBP group (Fig. 7E and F), indicating a superior long-term anti-inflammatory effect of NBP@Gel in vivo. LPS-induced BV2 cells in vitro demonstrated that both NBP and NBP@Gel could regulate microglial polarization towards the anti-inflammatory phenotype. Representative immunofluorescence images showed robust induction of iNOS in the LPS group, which was reduced by both NBP and NBP@Gel (Fig. 7G). Quantitative analysis confirmed a significant decrease in iNOS fluorescence intensity in both treatment groups, with NBP showing a stronger reduction than NBP@Gel (Fig. 7I). Representative staining of CD206 is shown in Fig. 7H, and quantification demonstrated that both NBP and NBP@Gel significantly altered CD206 fluorescence intensity compared with LPS (Fig. 7J), with NBP producing a larger change than NBP@Gel.

### 3.8 NBP@Gel enhances synaptic connection at day 14 after TBI

**Fig. 8:**
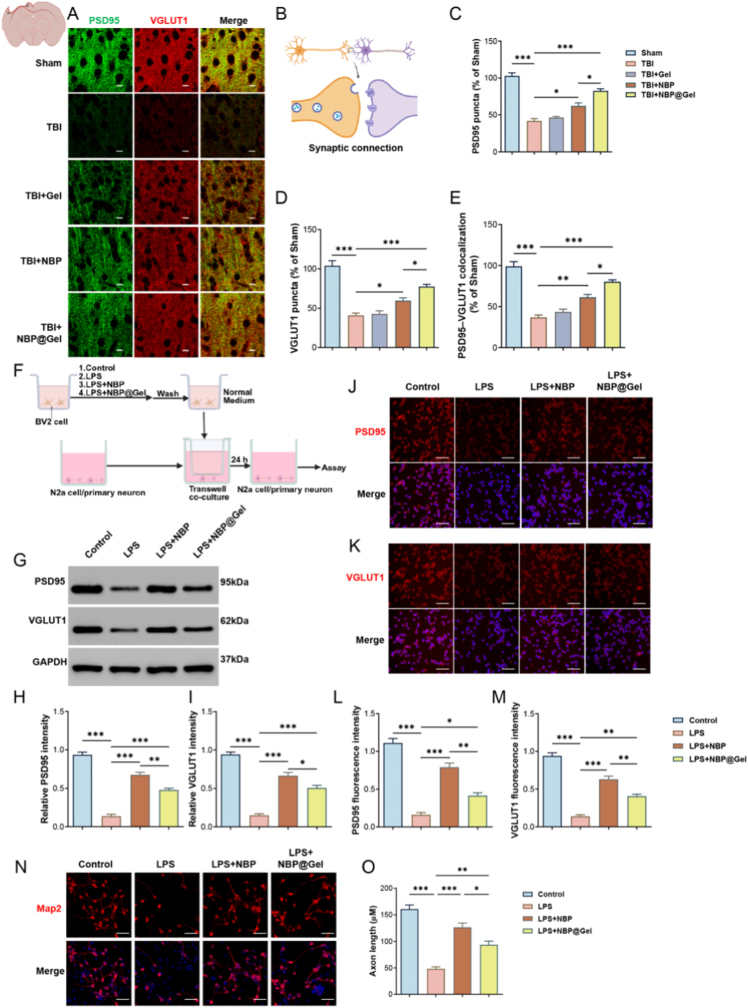
**NBP@Gel promotes synaptic connection during the chronic phase of TBI.** (A) Representative immunofluorescence images of synaptic markers PSD95 (green) and VGLUT1 (red) in peri-injury brain regions from the Sham, TBI, TBI + Gel, TBI + NBP, and TBI + NBP@Gel groups at day 14. Scale bars: 10 μm. (B) Schematic illustration of synaptic connection. (C) Quantification of PSD95 levels presented as percentage of Sham. (D) Quantification of VGLUT1 levels presented as percentage of Sham. (E) Quantification of PSD95-VGLUT1 colocalization presented as percentage of Sham. (F) Schematic illustration of the BV2 cells - N2a cells/primary neurons co-culture experiment. (G) Representative western blot bands of PSD95 and VGLUT1 in N2a cells. (H-I) Densitometric analysis of PSD95 (H) and VGLUT1 (I) protein expression normalized to GAPDH. (J-K) Representative immunofluorescence staining images of PSD95 (J) and VGLUT1 (K) in N2a cells. Scale bars: 100 μm. (L-M) Quantitative analysis of PSD95 (L) and VGLUT1 (M) fluorescence intensity in N2a cells. (N) Representative immunofluorescence staining images of MAP2 in primary neurons. Scale bars: 100 μm. (O) Quantification of axon length. All quantitative data are presented as mean ± SEM (C-E, n = 5 mice per group; H-I, L-M, and O, n = 3 independent experiments). Statistical significance was assessed by one-way ANOVA followed by Tukey's post hoc test. ∗P < 0.05, ∗∗P < 0.01, ∗∗∗P < 0.001. (For interpretation of the references to color in this figure legend, the reader is referred to the Web version of this article.)

We assessed whether NBP@Gel promotes synaptic connectivity by immunofluorescence staining labeling the postsynaptic marker PSD95 and the presynaptic glutamatergic marker VGLUT1 in the peri-injured cortex at day 14 after TBI. Representative immunofluorescence images of PSD95 and VGLUT1 in the peri-injured cortex are shown in Fig. 8A, with a schematic illustration of synaptic connection presented in Fig. 8B. Compared with Sham, the TBI group exhibited a marked reduction in PSD95 and VGLUT1 signals, along with decreased PSD95-VGLUT1 colocalization (Fig. 8A). Quantification further confirmed significant decreases in PSD95 puncta (Fig. 8C), VGLUT1 puncta (Fig. 8D), and PSD95–VGLUT1 colocalization (Fig. 8E) in the TBI group. While the TBI + Gel group showed limited improvement relative to TBI, both NBP and NBP@Gel increased PSD95 puncta, VGLUT1 puncta, and their colocalization compared with TBI, NBP@Gel produced a greater increase than NBP, indicating that NBP@Gel most effectively promotes synaptic connectivity among the treatments (Fig. 8A–E). To investigate whether NBP enhances synaptic connection via microglial polarization, BV2 cells were treated with LPS, NBP, or NBP@Gel, and the treated BV2 cells were then co-cultured with N2a cells to evaluate synaptic markers. WB analysis and densitometric quantification revealed LPS treatment of BV2 cells reduced the expression levels of PSD95 and VGLUT1, with both NBP and NBP@Gel increasing their levels (Fig. 8G–I). In parallel, representative immunofluorescence images showed that LPS-induced BV2 cells markedly reduced expression of PSD95 (Fig. 8J) and VGLUT1 in N2a cells (Fig. 8K) compared with control. Both NBP and NBP@Gel partially restored PSD95 and VGLUT1 in N2a cells when applied to BV2 cells.(Fig. 8J and K). This restoration was supported by fluorescence intensity quantification (Fig. 8L and M). To further validate the protective effect in a more physiologically relevant neuronal model, primary neurons were assessed by MAP2 immunostaining after indirect co-culture with BV2 cells. Representative images showed that LPS-induced BV2 cells markedly impaired neuronal morphology, whereas both NBP and NBP@Gel partially restored MAP2-positive neurite extension (Fig. 8N). Quantification of axon length confirmed that LPS-induced BV2 significantly reduced neuronal process length, while NBP and NBP@Gel both improved this parameter compared with the LPS group, with NBP showing a stronger effect than NBP@Gel under the current in vitro conditions (Fig. 8O). These findings support that NBP and NBP@Gel promote synaptic connection,via modulation of microglial polarization.

### 3.9 In vivo safety assessment indicates that NBP@Gel exhibits good biocompatibility

**Fig. 9:**
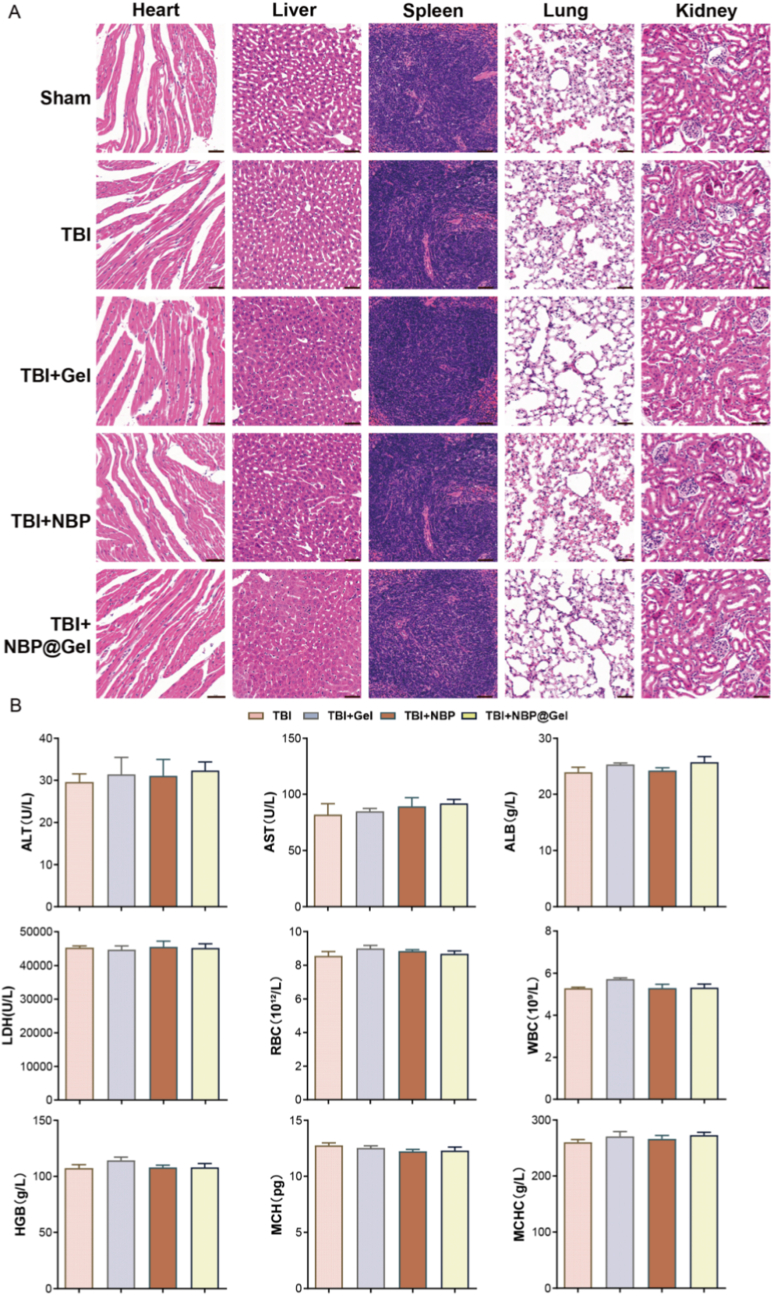
**In vivo biocompatibility evaluation of NBP@Gel.** (A) Representative H&E-stained images of major organs, including the heart, liver, spleen, lung, and kidney, from each group. Scale bar = 50 μm. (B) Blood biochemical and hematological parameters, including ALT, AST, ALB, LDH, RBC, WBC, HGB, MCH, and MCHC. Data are presented as mean ± SEM (n = 5 mice per group).

CCK-8 assays and Live/Dead staining demonstrated that NBP and NBP@Gel did not display cytotoxicity in N2a cells (Fig. S4B–C). Finally, we examined the systemic biocompatibility of NBP@Gel by assessing potential pathological changes in major organs using H&E staining. As shown in Fig. 9A, representative H&E images of the heart, liver, spleen, lung, and kidney from the Sham, TBI, TBI + Gel, TBI + NBP, and TBI + NBP@Gel groups displayed no obvious histomorphological abnormalities. All treatment groups showed organ structures that were intact and comparable to the Sham group, indicating that NBP@Gel implantation did not induce detectable systemic toxicity under the experimental conditions. In addition, systemic safety was assessed by blood biochemical and hematological analyses on day 14. ALT, AST, ALB, LDH, RBC, WBC, HGB, MCH, and MCHC showed no abnormal changes across the TBI, TBI + Gel, TBI + NBP, and TBI + NBP@Gel groups (Fig. 9B), supporting that NBP@Gel is biocompatible without detectable systemic toxicity.

## 4 Discussion

The absence of useful therapeutic targets and limitations of the underlying pathogenic mechanisms in TBI continue to be significant obstacles to better treatment. To address this gap, we constructed a multi-layer evidence chain linking iron metabolism to TBI prognosis and therapeutic vulnerability. Higher ferritin was inversely associated with survival, indicating ferritin as a clinically useful risk marker. The Kaplan-Meier and time-dependent ROC analyses corroborate ferritin as a prognostic indicator for TBI, aligning with Simon et al.'s findings that increased serum ferritin at admission correlates with poorer outcomes in severe TBI [27]. MR suggested a potential causal contribution of circulating iron to TBI risk. There were consistent SNP-level effects and robust sensitivity analyses (Fig. 1C–F). Experimental evidence consistently indicates that TBI induces iron deposition in the injured cortex, and that iron overload is associated with TBI and contributes to neuronal injury [28]. At the tissue level, transcriptomic profiling of a TBI dataset revealed coordinated enrichment of ferroptosis- and iron transport–related programs (Fig. 1G–J). Our transcriptome analysis revealed STEAP3 as a differentially expressed gene following TBI, aligning with the role of STEAP3 as an endosomal ferrireductase that reduces Fe^3+^ to Fe^2+^, hence facilitating lipid peroxidation and ferroptotic damage [29]. NCOA4 connects ferritin and ferroptosis on a molecular level by being the selective cargo receptor for ferritinophagy, which helps break down ferritin and liberate iron [30]. Past research has shown that the amount of NCOA4 mRNA may not change even when the amount of protein increases a lot [31]. In line with this regulatory approach, NCOA4 did not appear as a differentially expressed gene in our transcriptome study; nonetheless, Western blotting revealed a significant rise in NCOA4 protein following TBI (Fig. 1K), alongside raised STEAP3 levels. These upstream alterations were accompanied by a ferroptosis-related modification in xCT/ACSL4. Ferroptosis-driven lipid peroxidation is increasingly viewed as a key component of secondary injury after TBI, and experimental work shows that suppressing ferroptosis can reduce neuronal loss and improve functional outcomes [32]. Our findings establish a clinic-to-mechanism evidence chain implicating iron-linked ferroptosis in TBI.

Several studies further suggest that natural compounds can ameliorate TBI pathology by inhibiting lipid peroxidation and restoring antioxidant pathways,and a recent meta-analysis also supports beneficial effects of quercetin in TBI models [[33], [34], [35]]. NBP has also been shown to provide neuroprotection in TBI through multiple mechanisms, such as reducing oxidative damage through Nrf2-ARE signaling, stopping apoptotic pathways and NF-κB-linked neuroinflammation [15,36,37]. Building on these reports, we further investigated NBP and identified GSK-3β as an upstream regulatory node: network pharmacology highlighted GSK-3β as a central hub and docking supported a feasible interaction between NBP and GSK-3β (Fig. 2B–E). Mechanistically, GSK-3β is known to act upstream of Fyn to promote NRF2 nuclear export, thereby constraining NRF2-dependent antioxidant gene programs such as HO-1 and NQO1 [38]. In line with this mechanism, our results show that NBP modulates the GSK-3β–Fyn–NRF2 axis and enhances NRF2/HO-1/NQO1 while shifting ferroptosis-related proteins toward favorable levels (Fig. 3A–K). Activation of GSK-3β with DIF-3 or inhibition of NRF2 with ML385 partially blunted the anti-ferroptotic and antioxidant effects of NBP. Oxidative lipid injury markers (e.g., 4-HNE and MDA) rise early after TBI and are commonly used to track lipid peroxidation burden [[39], [40], [41]]. Accordingly, in vivo we observed 4-HNE elevation at day 3 post-TBI that was significantly reduced by both NBP and NBP@Gel (Fig. 6A and B), and tissue MDA/SOD assays further supported attenuation of oxidative injury at both day 3 and day 14 (Fig. 6C and D), with NBP@Gel showing a clearer advantage at the later time point. Although these pharmacological perturbation experiments strengthen the mechanistic support beyond correlative observation, additional ferroptosis-specific rescue experiments or genetic manipulation of key regulators would be valuable to provide stronger causal validation in future studies.

In most previous studies, NBP has been administered via intravenous or intraperitoneal injection [42,43]. However, these delivery routes face several limitations, including limited drug accumulation in the injured brain due to peripheral distribution and rapid clearance [44,45], the spatially and temporally heterogeneous blood–brain barrier disruption after TBI that makes intracerebral exposure difficult to predict and dose-optimize [46], and a short effective duration that requires frequent administration and reduces treatment compliance [47]. Similar challenges have been reported for other systemically delivered nanoformulations or lipid-based carriers, despite targeting strategies, are largely sequestered by the reticuloendothelial system—particularly the liver and spleen—thereby restricting effective drug delivery to the brain [44,48]. Cortical contusion and hematoma evacuation often leave a surgically accessible cavity, which provides a site for local implantation of injectable materials that can fill the cavity and match brain tissue mechanics. An increasing number of studies have employed injectable hydrogels for localized administration in TBI [20,25,49,50]. For example, ROS-responsive in situ–forming hydrogels have been reported to inhibit oxidative stress and neuroinflammation and thereby improve TBI repair, highlighting the value of local biomaterial intervention in the early stage [51]. Consistent with this concept, our NBP@Gel achieved effective acute neuroprotection: at day 3 after TBI, NBP@Gel significantly reduced lipid peroxidation and improved antioxidant indices, reaching an efficacy comparable to free NBP in key ferroptosis/oxidative stress–related readouts (Fig. 6A–D). Meanwhile, many biomaterial approaches mainly target early injury processes and provide limited support beyond the acute stage, whereas long-term recovery requires sustained microenvironment modulation and functional reconstruction. The biocompatibility and brain-matching mechanisms of injectable compounds are essential for intracranial application. In our investigation, NBP@Gel demonstrated favorable biocompatibility, as no significant histopathological abnormalities were detected in major organs post-implantation (Fig. 9A), and no obvious abnormal changes were observed in blood biochemical or hematological parameters (Fig. 9B). Furthermore, the pre-gel solutions demonstrated excellent injectability and could be effortlessly administered by a syringe needle, facilitating conformal filling of uneven lesion cavities (Fig. 4D–F). From a mechanical perspective, our NBP@Gel demonstrated viscoelasticity that closely aligned with the modulus range of brain tissue. The storage modulus of the hydrogel was quantified to be between 250 and 550 Pa, consistent with the documented values for human brain tissue, especially gray matter, which exhibits a shear modulus of between 100 and 1000 Pa [52]. Extending local drug availability is particularly valuable during the subacute-to-chronic phase of TBI, when repair is increasingly dominated by extracellular-matrix and tissue remodeling processes [53,54]. Recent studies further indicate that functional injectable biomaterials can promote tissue regeneration not only by sustaining local release of therapeutic agents or bioactive signals, but also by actively modulating the injury microenvironment through multiple interactions, including maintaining neuronal survival and function and promoting angiogenesis [55,56]. From a design perspective, in situ–forming depot systems have been widely developed in non-neural tissues to prolong local drug exposure [57]. The high viscosity and hydrophobicity of SAIB form a dense matrix that slows drug diffusion, creating a physical barrier that results in controlled, sustained release over time [58]. Motivated by this depot concept and the evolving pathology of TBI, we integrated cyclodextrin inclusion (to address poor solubility of NBP) with SAIB depot (to prolong retention and support delayed release), thereby enabling a staged delivery profile aligned with acute-to-chronic pathological needs (Fig. 4D–K). Consistent with a staged-release design, NBP@Gel showed an initial burst within the first day (∼56% cumulative release), followed by a slower sustained phase reaching ∼60% by day 3 and ∼70% by day 7 (Fig. 4K). This “burst + sustained” profile is well suited to provide early dosing in the acute phase while maintaining prolonged exposure during the subsequent repair phase. This stage-dependent release pattern is consistent with the intended therapeutic logic of the hydrogel system: early burst release supports acute anti-ferroptotic neuroprotection, whereas sustained local exposure is more relevant to prolonged modulation of microglial phenotype and synaptic remodeling.

Beyond acute ferroptosis control, durable functional recovery after TBI requires preservation of neuronal integrity and reconstruction of neuroimmune and synaptic homeostasis. Previous studies emphasize that the secondary injury cascade extends well beyond the acute window, and that sustained local modulation of the lesion microenvironment is often necessary to support long-term functional repair [53,59]. Similar stage-dependent principles have been highlighted in stroke recovery, where intermittent theta burst stimulation improved post-stroke recovery by modifying microglial state to support neuroplasticity, and melatonin and baicalein were found to provide neuroprotection in part by limiting pro-inflammatory microglial polarization [[60], [61], [62]]. Guided by the evolving pathology of TBI, our hydrogel system was designed to provide staged local delivery, enabling an early protective dose during the acute phase and sustained lesion-site exposure during the subsequent repair phase. Mechanistically, this design is supported by the temporal dynamics of post-TBI neuroinflammation: microglia/macrophages exhibit an early, transient increase in anti-inflammatory features, followed by a decline of this reparative phenotype and persistence of pro-inflammatory signaling over the subacute-to-chronic period, highlighting the need for prolonged microenvironment modulation rather than a purely acute intervention [50]. In vivo behavioral assays showed that NBP@Gel improved motor coordination and balance over time, and also enhanced cognitive function at the later stage, with a more evident advantage compared with NBP (Fig. 5B–D), consistent with the concept that sustained lesion-site exposure better matches the prolonged secondary injury timeline. NBP@Gel reduced tissue disruption and neuronal loss in pericontusional regions, as seen in H&E and Nissl stains, and increased surviving neuron density at day 14 (Fig. 5F–H). This indicates direct neuroprotection during chronic phase. In parallel, evidence suggests that microglial phenotypes and cytokines are key determinants of neural repair, with pro-inflammatory activation exacerbating damage while anti-inflammatory programs support resolution and regeneration [63,64]. Our Iba1^+^CD16^+^ and Iba1^+^CD206^+^ analyses revealed that NBP@Gel more effectively suppressed the pro-inflammatory microglial signature while maintaining a higher density of anti-inflammatory microglia at day 14, alongside higher TGF-β and IL-10 levels (Fig. 7A–F). BV2 assays further supported that both NBP and NBP@Gel modulate inflammatory marker expression in vitro (Fig. 7G–J). Importantly, this does not contradict the in vivo findings. In the lesion environment after TBI, therapeutic benefit depends not only on immediate pharmacodynamic intensity, but also on whether sufficient local drug exposure can be maintained across the evolving pathological timeline. In this setting, in situ implantation of NBP@Gel is expected to sustain drug exposure in peri-injury tissue, thereby supporting prolonged immunomodulation and tissue repair processes. Synaptic disruption is another recognized mechanism underlying persistent functional deficits after TBI, and microglial polarization can modulate synaptic plasticity and synaptic remodeling, making restoration of synaptic connections an increasingly important readout of meaningful circuit-level recovery [65]. Synaptic connection was reflected by increased PSD95 and VGLUT1 signals and improved PSD95-VGLUT1 co-localization at day 14 in vivo (Fig. 8A–E), while in vitro experiments confirmed that NBP and NBP@Gel elevated synaptic marker expression at both staining and protein levels (Fig. 8G–M). Additional primary neuron studies further support that NBP and NBP@Gel can restore neuronal morphology and neurite extension under inflammatory conditions (Fig. 8N and O). Collectively, our data suggest that NBP@Gel offers a lesion-matched, multi-stage therapeutic profile—early attenuation of ferroptosis injury followed by sustained synaptic connection—providing a practical framework for “full-course” TBI intervention.

## 5 Conclusion

We developed an injectable NBP@Gel hydrogel for in situ implantation after TBI. This hydrogel enables full-course treatment by inhibiting early ferroptosis and later supporting neuronal and synaptic plasticity. These features may facilitate future translation. It provides sustained, localized drug delivery, addressing the challenge of treating both acute and chronic phases of TBI. These findings suggest that the therapeutic advantage of NBP@Gel lies not in combining multiple pharmacological agents, but in enabling a single drug to exert multi-target effects across different stages of TBI, including anti-ferroptotic protection in the acute phase, immunomodulatory action in the subacute phase, and support for synaptic remodeling and functional recovery at later stages. From a translational perspective, this design highlights the potential value of a strategy based on single administration throughout the disease course, in which one locally delivered drug may provide staged therapeutic benefits across the evolving pathology of TBI. However, several issues still need to be addressed: (i) long-term biocompatibility, with further evaluation required to assess potential immune responses and systemic effects; (ii) more comprehensive and multidimensional assessment strategies, including advanced behavioral testing, signaling pathway interrogation, and imaging techniques to monitor hydrogel distribution and tissue regeneration; and (iii) more precise control of drug release, focusing on optimizing the release curve for more predictable and sustained exposure. Addressing these challenges will be essential for successful clinical translation of NBP@Gel as a promising therapeutic strategy for TBI.

## Ethical approval

All in vivo procedures were approved by the Animal Ethical Committee of Beijing Rehabilitation Hospital, Capital Medical University (approval ID: 2024bkky-087), and were conducted in accordance with relevant institutional guidelines and national regulations for the care and use of laboratory animals.

## CRediT authorship contribution statement

**Changbin Hu:** Formal analysis, Validation, Visualization, Writing – original draft. **Ning Li:** Conceptualization, Methodology, Supervision, Writing – review & editing. **Yuanyuan Ran:** Funding acquisition, Methodology, Supervision. **Hao Fu:** Methodology, Resources. **Lei Zhou:** Resources, Software. **Fanglei Li:** Conceptualization, Data curation. **Chenye Qiao:** Validation, Visualization. **Chuhan Liu:** Formal analysis, Methodology. **Guiqin Tian:** Investigation, Visualization. **Guoping Guan:** Conceptualization, Data curation. **Wei Su:** Formal analysis, Supervision. **Jianing Xi:** Data curation, Methodology. **Zongjian Liu:** Conceptualization, Funding acquisition, Project administration, Writing – review & editing.

## Declaration of competing interest

The authors declare no competing financial interests.

## Data Availability

Data will be made available on request.
