# Supplementary material for: Sequential release of N-butylphthalide via multifunctional hydrogel for rapid neuroprotection and sustained neural repair after traumatic brain injury
Source: Mater Today Bio. 2026 Jul 6;39:103436. doi: 10.1016/j.mtbio.2026.103436 (PMC13356776; doi:10.1016/j.mtbio.2026.103436)
Supplement: Multimedia component 1 [file mmc1.docx]

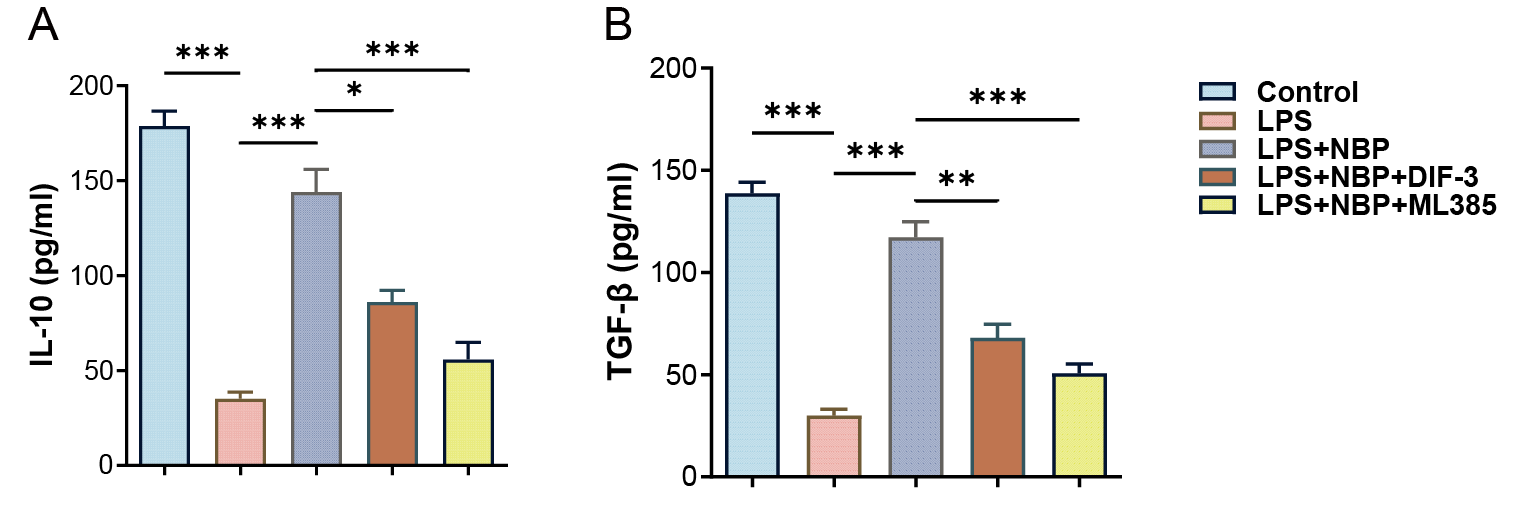


Figure S1. NBP enhances anti-inflammatory cytokine production in LPS-stimulated BV2 cells. (A) IL-10 level measured by ELISA. (B) TGF-β level measured by ELISA. All quantitative data are presented as mean ± SEM (n = 3 independent experiments). Statistical significance was assessed by one-way ANOVA followed by Tukey’s post hoc test. *P < 0.05, **P < 0.01, ***P < 0.001.


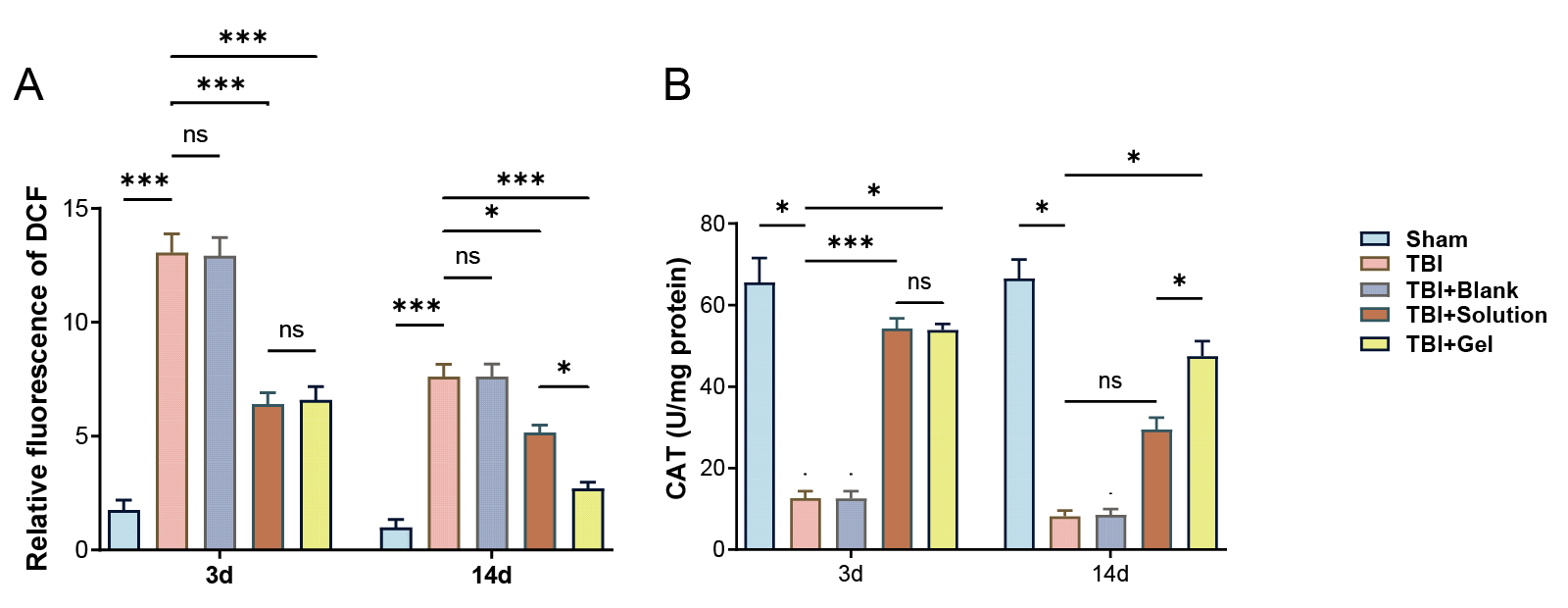


Figure S2. NBP@Gel alleviates ROS and enhances CAT activity in peri-lesional brain tissue after TBI. (A) ROS level assessed by DCF fluorescence; (B) CAT activity (U/mg protein). All quantitative data are presented as mean ± SEM (n = 5 mice per group). Statistical significance was assessed by one-way ANOVA followed by Tukey’s post hoc test. ns, not significant; *P < 0.05, ***P < 0.001.


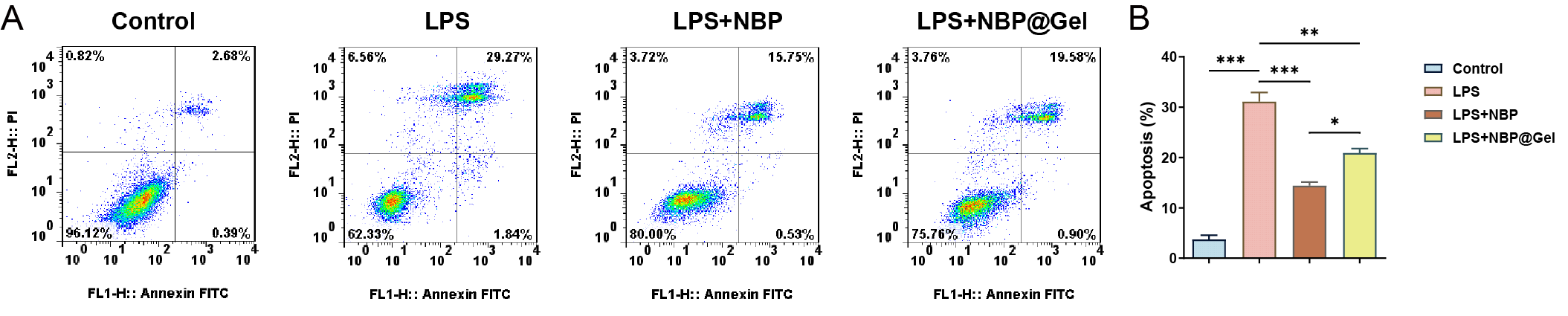


Figure S3. NBP and NBP@Gel reduce apoptosis in co-cultured N2a cells. (A) Representative Annexin V-FITC/PI flow cytometry plots; (B) Quantification of apoptosis rate. All quantitative data are presented as mean ± SEM (n = 3 independent experiments). Statistical significance was assessed by one-way ANOVA followed by Tukey’s post hoc test. *P < 0.05, **P < 0.01, ***P < 0.001.


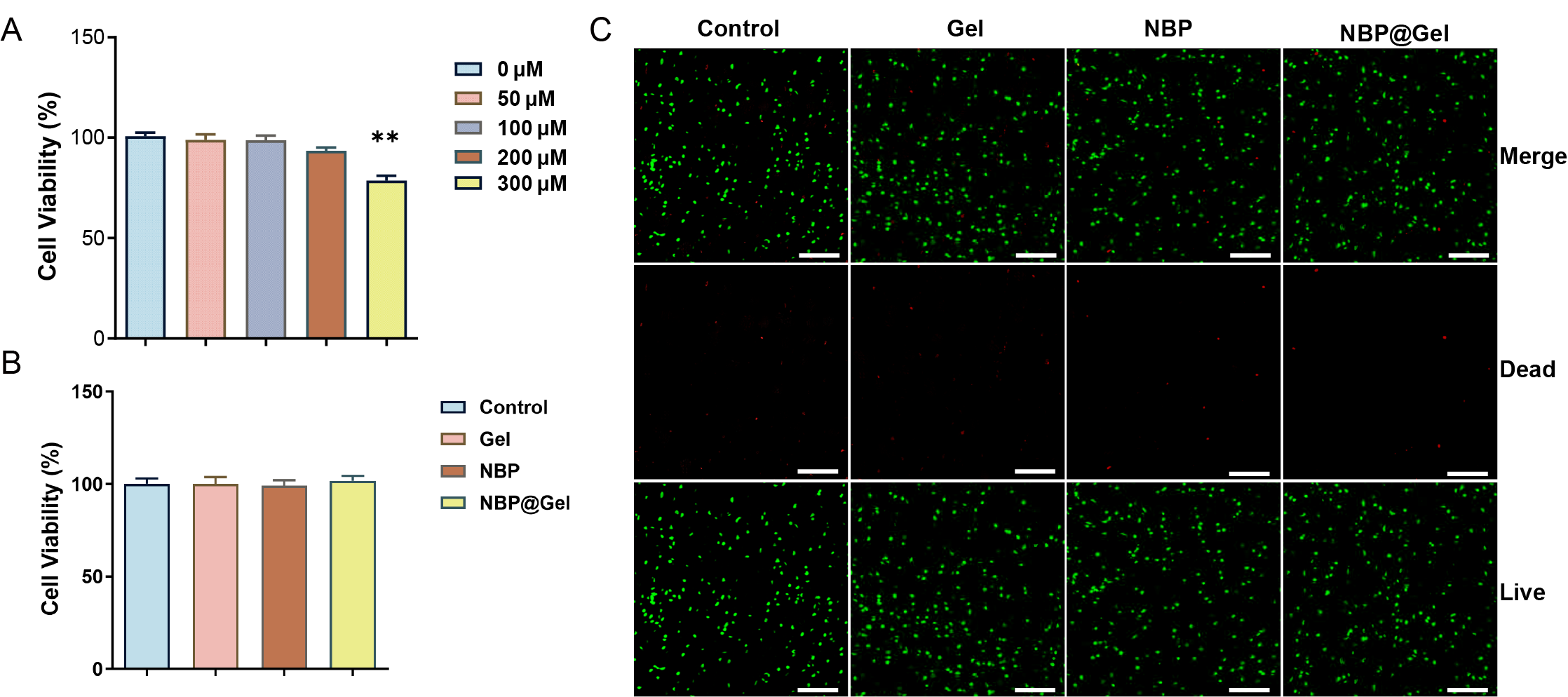


Figure S4. Gel and NBP@Gel exhibit good cytocompatibility in vitro. (A) Cell viability of N2a cells treated with different concentrations of NBP (0, 50, 100, 200, and 300 μM), as measured by CCK-8 assay. (B) Cell viability of N2a cells treated with Gel extract, free NBP (200 μM), or NBP@Gel extract (200 μM equivalent in NBP@Gel) for 24 h. (C) Representative live/dead staining images of N2a cells after different treatments. Live cells are shown in green, and dead cells are shown in red. Scale bars=100 μm. All quantitative data are presented as mean ± SEM (n = 3 independent experiments). Statistical significance was assessed by one-way ANOVA followed by Tukey’s post hoc test. **P < 0.01 compared with 0 μM group.


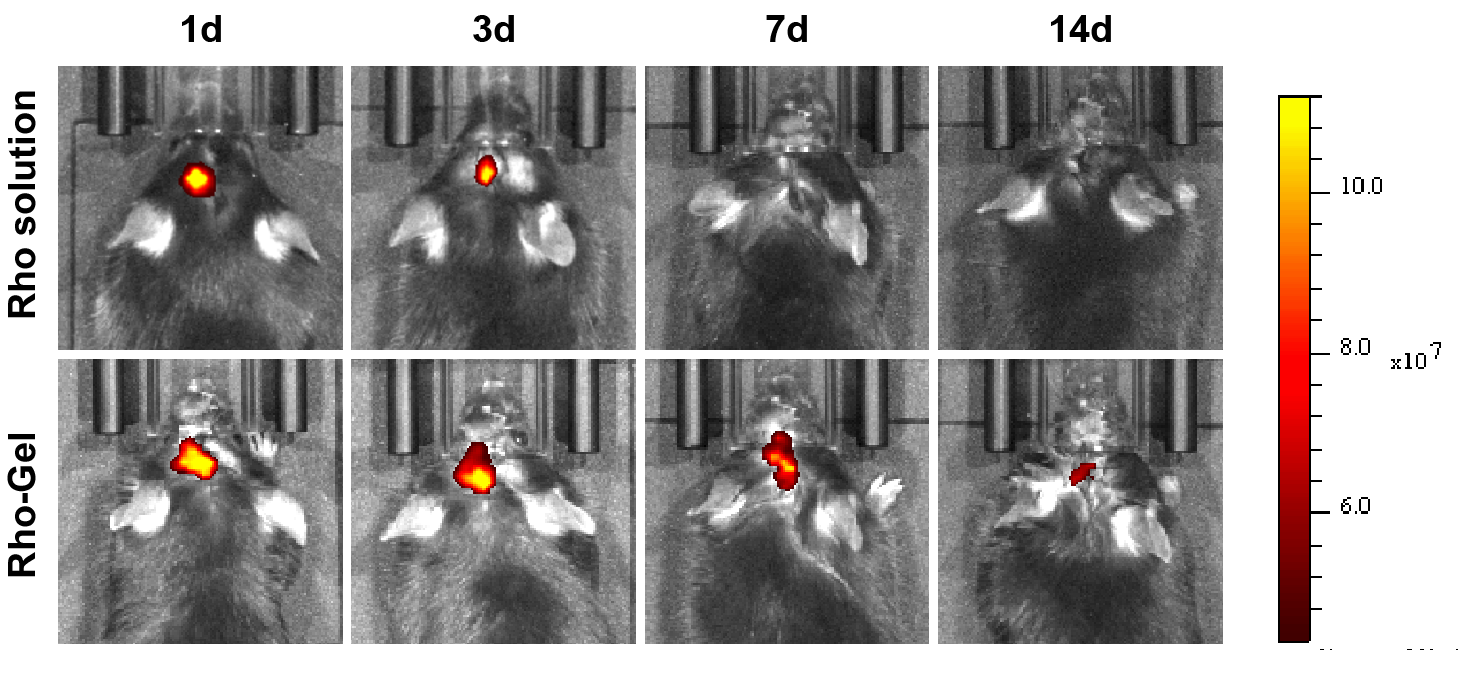


Figure S5. In vivo fluorescence tracking of hydrogel degradation. Rhodamine B-loaded hydrogel (Rho-Gel) or free Rhodamine B solution (Rho Solution) was implanted into the lesion site after TBI. Fluorescence signals were monitored using a small-animal imaging system (IVIS® Lumina III, PerkinElmer, USA) at 1, 3, 7, and 14 days post-implantation. Representative images are shown.
